# Halo- and Thiocarbazomycins from Coral- and Coral Reef Sands-Derived *Actinomycetes*

**DOI:** 10.3390/md20080537

**Published:** 2022-08-21

**Authors:** Qiaoling Wu, Hongjie Zhu, Changli Sun, Le Zhou, Huimin Wang, Songbiao Shi, Xinpeng Tian, Jianhua Ju

**Affiliations:** 1 Southern Marine Science and Engineering Guangdong Laboratory (Guangzhou), Guangzhou 511458, China; 2 CAS Key Laboratory of Tropical Marine Bio-Resources and Ecology, RNAM Center for Marine Microbiology, Guangdong Key Laboratory of Marine Materia Medica, South China Sea Institute of Oceanology, Chinese Academy of Sciences, Guangzhou 510301, China; 3 University of Chinese Academy of Sciences, Beijing 110039, China; 4 School of Pharmaceutical Sciences, Shandong University, Jinan 250012, China; 5 School of Pharmacy, Institute of Marine Drug, Guangxi University of Traditional Chinese Medicine, Nanning 530200, China

**Keywords:** carbazole, alkaloids, coral-associated actinomycetes, halogen compound, natural products

## Abstract

Four actinomycete strains isolated from the coral *Acropora austera* and coral sand samples from the South China Sea, were found to produce a series of halogenated compounds baring similar ultraviolet absorption based on the analysis of HPLC and LC-MS. The production titers of halogenated compounds from *Streptomyces diacarni* SCSIO 64983 exceeded those of other similar strains leading us to focus on SCSIO 64983. Four new thiocarbazomycins A–B (**1**–**2**), chlocarbazomycin E (**3**), and brocarbazomycin A (**4**), together with three known chlocarbazomycins A–C (**5**–**7**) containing a carbazole core were identified, and their structures were determined using a combination of spectroscopic analysis including HRESIMS, 1D and 2D NMR. Structurally speaking, compounds **1** and **2** have the rare sulfur-containing carbazole nuclei, and **3** and **4** contain Cl and Br atoms, respectively. Although these compounds have not yet been found to have obvious biological activity, their discovery highlights the role of molecular libraries in subsequent drug discovery campaigns.

## 1. Introduction

In marine environments symbiotic relationships between corals and coral-associated bacteria (CAB) are essential for host organism homeostasis. Many CABs can prevent coral diseases and protect corals from pathogens by producing defensive agents with antiviral, anti-bacterial, anti-fouling, and anti-parasitic activities [1,2,3]. Coral-derived actinomycetes have the potential to produce polyketides [4,5,6,7,8], alkaloids [9], and terpenes [10], etc.; all are important sources for drug discovery and development [11]. In particular, carbazole compounds have a wide range of biological activities, including but not limited to antibacterial, anti-platelet aggregation, anti-inflammatory, anti-tumor, and neuroprotective activities [12,13]. Graebe and Glaser first discovered compounds possessing a carbazole nucleus from coal tar in 1872; subsequent efforts found that some carbazole-derived compounds could be isolated from roots, root barks, cow urine, and cyanobacteria of higher plants [12,13]. There are, however, very few reports of carbazole compounds being produced by marine microorganisms.

Four strains—SCSIO 64983, SCSIO 68034, SCSIO 68036, and SCSIO 68060—were identified as *Streptomyces diacarni* by 16S rRNA sequence analyses following their isolation from *Acropora austera* and coral reef sand samples. Strain fermentation/growth conditions were optimized using a variety of media, their fermentation products were analyzed by HPLC and LC-MS, and a class of small molecules with similar ultraviolet absorption was discovered. In light of these early findings, we chose to focus on *S. diacarni* SCSIO 64983. This strain demonstrated optimum titers of seven compounds with sulfur or halogen substitutions including chlocarbazomycins A–C (**5**–**7**) [13]; four of the seven SCSIO 64983 products were new compounds: the thiocarbazomycins A–B (**1**–**2**), chlocarbazomycin E (**3**), and brocarbazomycin A (**4**). Moreover, once in hand, we evaluated the antibacterial activities of the SCSIO 64983-derived agents against 14 different pathogenic bacteria using a disk diffusion method.

## 2. Results

### 2.1. Strain Isolation and Identification

**Figure 1:**
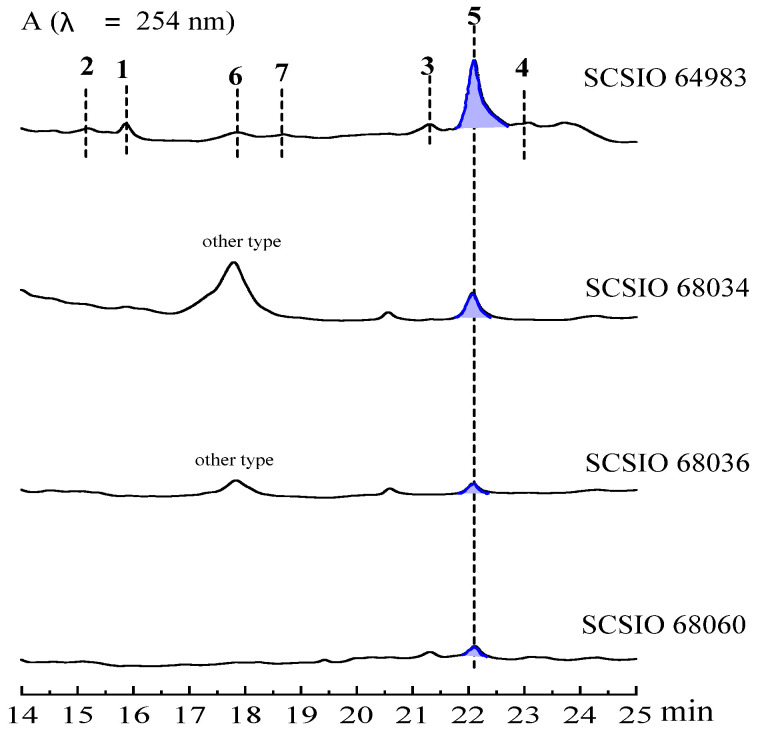
Metabolite analyses of the N4 medium fermentation products of four coral-related *Actinomycetes* (the ratio of the peak area of compound **5** in the above four strains is about 1:44%:11%:9%).

**Figure 2:**
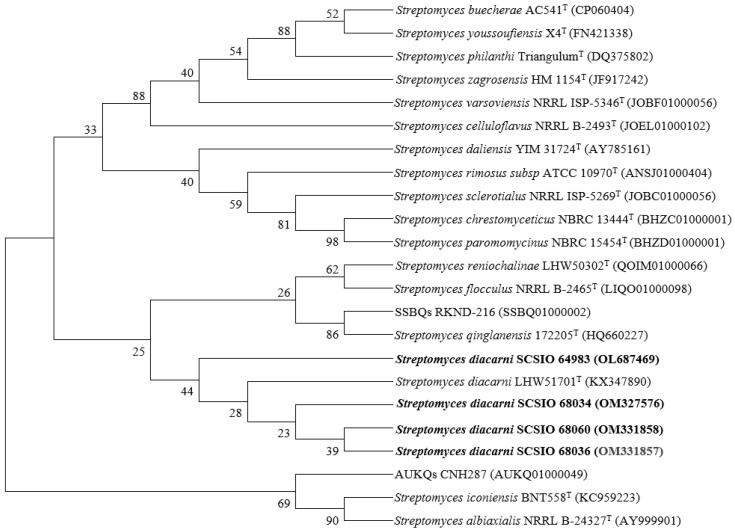
The phylogenetic tree of the four coral- and coral reef sand-derived *Actinomycetes*.

The coral *Acropora austera* and coral reef sand samples were collected at a water depth of 20 meters in the Zhongsha Islands, South China Sea. The collected samples were surface-disinfected, and then aseptically ground to obtain the original solution, which was diluted 100 times and spread onto ISP2 and 2216E media, respectively. Following incubation at 28 °C for one week, the colonies were picked and purified continuously. The obtained strains were fermented to eliminate duplication, and the metabolite analyses of N4 medium revealed that strains SCSIO 64983, SCSIO 68034, SCSIO 68036, and SCSIO 68060 produced a class of compounds with the same UV absorption at 254 nm (Figure 1). These strains were all identified as *Streptomyces diacarni* on the basis of 16S rRNA sequencing and morphological analysis [14]. We then employed the neighbor-joining method to construct a phylogenetic tree using MEGA7.0.14 software (Figure 2). *S. diacarni* SCSIO 64983, because of its relatively high yields and rich varieties of compounds, was selected as the primary focus of the current study. 

### 2.2. Structure Elucidation

Comparisons of the NMR and HRESIMS data sets reported in the literature [13] to the data generated herein revealed that compounds **5**, **6,** and **7** are the previously known compounds chlocarbazomycin A, chlocarbazomycin B, and chlocarbazomycin C, respectively.

**Figure 3:**
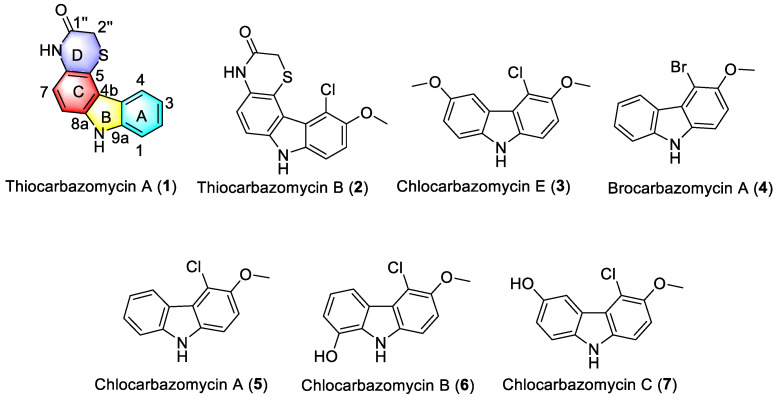
Structures of compounds **1**–**7** isolated from *S. diacarni* SCSIO 64983.

**Figure 4:**
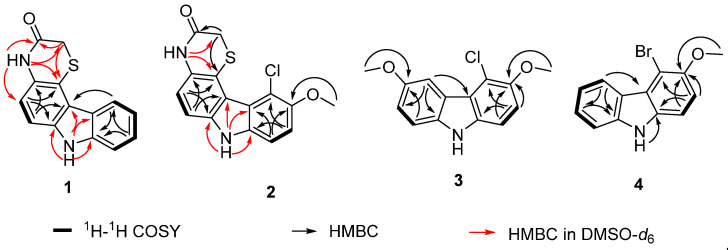
^1^H-^1^H COSY and key HMBC correlations for compounds **1**–**4.**

With respect to SCSIO 64983-derived compounds not clearly assignable as established agents, thiocarbazomycin A (**1**) was identified as a light brown powder with molecular formula C_14_H_10_N_2_OS and 11 degrees of unsaturation as deduced from HRESIMS data (Figure S11). The ^13^C NMR and DEPT135 spectra of **1** revealed 14 carbon signals which were assigned to seven quaternary carbons (*δ*_C_ 130.6, 115.2, 120.9, 123.8, 142.1, 138.2, and 167.1), six methine carbons (*δ*_C_ 110.1, 117.0, 123.6, 119.7, 126.8, and 111.8), and one methylene group (*δ*_C_ 30.6) (Table 1 and Table S1). The ^1^H NMR spectrum indicated the presence of six aromatic protons, and ^1^H-^1^H COSY spectrum of **1** revealed the presence of two spin systems: H-7 (*δ*_H_ 7.07)/ H-8 (*δ*_H_ 7.30) and H-1 (*δ*_H_ 7.45)/ H-2 (*δ*_H_ 7. 39) / H-3 (*δ*_H_ 7.17) / H-4 (*δ*_H_ 8.36), indicating that **1** contained at least two benzene rings. The HMBC correlations of H-8 (*δ*_H_ 7.30)/C-6 (*δ*_C_ 130.6, C), C-4b (*δ*_C_ 120.9, C) and H-7 (*δ*_H_ 7. 07)/C-5 (*δ*_C_ 115.2, C), C-8a (*δ*_C_ 138.2, C) suggested the presence of a 1,2,3,4-tetrasubstituted benzene ring (C ring). In addition, HMBC correlations of H-4 (*δ*_H_ 8.36) /C-9a (*δ*_C_ 142.1, C), and H-1 (*δ*_H_ 7.45) /C-3 (*δ*_C_ 119.7, CH), C-4a (*δ*_C_ 123.8, C) indicated the existence of a 1,2-disubstituted benzene ring (A ring). Two parts (A and C rings) appeared to be connected via C4a-C4b and C8a-NH-C9a, as suggested by HMBC correlations of H-4 (*δ*_H_ 8.36) /C-4b (*δ*_C_ 120.9, C), and 9-NH (*δ*_H_ 11.37) /C-4b (*δ*_C_ 118.8, C), C-4a (*δ*_C_ 121.9, C), C-9a (*δ*_C_ 140.2, C) and C-8a (*δ*_C_ 135.9, C); these findings were consistent with the presence of a pyrrole ring (B ring). HMBC correlations of 10-*N*H (*δ*_H_ 10.52)/ C-1” (*δ*_C_ 164.3, C) and C-2” (*δ*_C_ 29.0, CH) and the chemical shift of C-1” indicated the existence of a -CH_2_-CO-NH- linkage as well. HMBC correlations of H-2” (*δ*_H_ 3.59) to C-5 (*δ*_C_ 112.6, C), and 10-*N*H (*δ*_H_ 10.52)/ C-7 (*δ*_C_ 115.9, CH) and C-5 (*δ*_C_ 112.6, C), in conjunction with the remaining point of unsaturation and S atom indicated identity of the D ring. Therefore, the structure of compound **1**, hitherto named thiocarbazomycin A (**1**), was established (Figure 3 and Figure 4).

Thiocarbazomycin B (**2**), presented as a light brown solid with a molecular formula of C_15_H_11_ClN_2_O_2_S based on its ^13^C NMR and HRESIMS data (Figure S23). The NMR data for **2** are shown in Table 1. The combined HRESIMS and NMR data set suggested that **2** contained one methoxy group, one chlorine atom, one sulfur atom, one carbon-based group, two *N*-containing active hydrogens, 9 quaternary carbons, 4 methine units, and 1 methylene group. The existence of one chlorine atom in compound **2** could be deduced from its HRESIMS data corresponding to the pseudo-molecular peaks for [M + H]^+^ at 319.0303 and 321.0275 with a ratio of 3:1 as shown in Figure S23. The signals representing the two *N*-containing active hydrogens were shown in Table S1. The subunits indicated by 1D and 2D NMR data as shown in Figure 3 revealed that this compound is very similar to compound **1**, in that both are carbazole compounds. Compound **2** was found to have one more chlorine atom and one more methoxy group (*δ*_H_ 3.90, s) than compound **1**, and HMBC correlations of H-2/C-4 (*δ*_C_ 116.7) and 3-OCH_3_ /C-3 (*δ*_C_ 150.8) indicated that the chlorine atom and -OCH_3_ are attached to the C-4 and C-3 positions, respectively. Therefore, the structure of compound **2** was elucidated as thiocarbazomycin B (**2**).

Chlocarbazomycin E (**3**), also was found to be a light brown amorphous powder, and was found to have a molecular formula of C_14_H_12_ClNO_2_ by analysis of its HRESIMS data (Figure S31) and ^13^C NMR data. It was determined that **3** possesses nine degrees of unsaturation. ^1^H NMR showed that there are five hydrogens in the aromatic region, and detailed interpretation of its 1D NMR data (Table 1) suggested a high degree of structural similarity to chlocarbazomycin C (**7**) [13]. It was noted that spectroscopy of compound **3** displayed an additional signal of a methoxyl group which is absent in 7. Indeed, the HMBC correlation of 6-OCH_3_ (*δ*_H_ 3.89, s)/C-6 (*δ*_C_ 153.0) indicated the regiochemistry of the additional methyl group as shown in Figure 3. Thus, the structure of **3** was determined, and named chlocarbazomycin E.

Brocarbazomycin A (**4**) was isolated as a light brown solid and combined HRESIMS data (Figure S39) and NMR data revealed its molecular formula to be C_13_H_10_BrNO. The ^1^H and ^13^C NMR spectroscopic data of compound **4** were very similar to those of chlocarbazomycin A (**5**) [13], but the chemical shift for C-4 (*δ*_C_ 106.3) was found to be significantly up-field shifted in **4** (*δ*_C_ 117.1 in **5**). Combined with our knowledge of its molecular composition and origins, we proposed that a Cl-atom in **5** may simply, in **4**, be replaced by a Br-atom. This speculation was secured by the HMBC correlation of H-2/C-4 and HRESIMS profiling with a ratio of 1:1 between ^79^Br:^81^Br. Thus, the structure of **4** was readily determined, and named brocarbazomycin A.

### 2.3. Antibacterial Activity

Compounds **1**–**7** were evaluated for antibacterial activity using a disk diffusion method. The test pathogens used in this experiment were: 7 Gram-positive pathogens including *Staphylococcus aureus* ATCC 29213, *Staphylococcus aureus* Sau 1862, *Bacillus thuringiensis*, *Enterococcus faecalis* ATCC 29212, MRSA/methicillin-resistant *Staphylococcus aureus*, *Micrococcus luteus*, and *Bacillus subtilis*; and 7 Gram-negative bacteria including *Escherichia coli*, *Escherichia coli* ATCC 25922, *Klebsiella pneumoniae* ATCC 13883, MRPA/*Pseudomonas aeruginosa* pae 1873, *Vibrio alginolyticus* XSBZ14, *Acinetobacter baumannii* ADR-2, and *Acinetobacter baumannii* ATCC 19606. Unfortunately, we found that these compounds have no obvious inhibitory activity against the above pathogens. The diameter of their inhibition zone is about 7 mm, but the diameter of the filter paper is 6.5 mm. Therefore, we did not carry out the MIC (minimum inhibitory concentration) experiment.

## 3. Discussion

In this study, we demonstrated that a variety of coral-associated actinomycetes have ability to produce halocarbazomycins which may act as regulatory or other active molecules for these microorganisms or corals. Finally, seven halo- and thiocarbazomycins were isolated from *S. diacarni* SCSIO 64983, among which thiocarbazomycins A–B (**1**–**2**), chlocarbazomycins E (**3**), and brocarbazomycin A (**4**) were novel compounds. Comparisons with chlocarbazomycins A–D first reported by Lin [13], reveal that compounds **5**–**7** have the same structure as chlocarbazomycins A–C, respectively. Particularly noteworthy is that compounds **1** and **2** are S-substituted nitrogen heterocycles, the first of their kind to our knowledge. In addition, **4** has a bromide substitution; thus, **1**, **2**, and **4** are substantively different from previously reported tricyclic carbazoles [13]. In addition, we have to point out that, thus far, we have been unable to establish what kind of biological activities these agents may express. We have found however, that these compounds do not appear to express any significant antibacterial activities against the organisms employed herein. The chemical structures of halo- and thiocarbazomycins from *S. diacarni* SCSIO 64983 are unusual and may express interesting biological activities, though we have not yet identified them. 

## 4. Materials and Methods

### 4.1. General Eexperimental Procedures

Using bacteriological peptone, technical agar powder, malt extract powder (Guangdong Huankai Microbial Technology Co., Ltd., Guangzhou, China), and conventional materials such as glucose, sodium chloride, and magnesium sulfate (Guangzhou Chemical Reagent Factory, Guangzhou, China), sea salt (Guangdong Salt Industry Group Co., Ltd., Guangzhou, China), and other ingredients, strains were fermented and cultivated using an MQD-B1NR Minquan shaking incubator (Guangzhou Hezhong Biotechnology Co., Ltd., Guangzhou, China). Extracts were concentrated with a Laborota 4000 efficient rotary evaporator (Heidolph). Methanol, ethyl acetate, petroleum ether, and other analytically pure conventional chemical reagents (Guangzhou Chemical Reagent Factory, Guangzhou, China) were used for normal phase silica gel column chromatography (CC) with a size of 100–200 mesh silica (Jiangpeng Silica gel development, Inc., Shandong, China). Preparative chromatography grade acetonitrile (Beijing Bailingwei Technology Co., Ltd., Beijing, China ) was used to analyze samples with a high-pressure liquid chromatograph Infinity 1260 (Agilent Company, Palo Alto, CA, USA) on Phenomenex C18 column (150 mm × 4.6 mm), as well as for separation and purification on a Basic C18 semi-preparative column (10 mm × 250 mm, 5 μm) (Beijing Sepreis Technology) Co., Ltd., Beijing, China). A Bruker AVANCE 700/175 superconducting nuclear magnetic resonance spectrometer (Bruker, Bayern, Germany) was used to obtain NMR data. A Bruker maXis high resolution time-of-flight mass spectrometer (Bruker, Bayern, Germany) was used to obtain the HR ESIMS data. The crystal structure of compound **5** was analyzed using an XtaLABPro *X*-Ray single crystal diffractometer (Rigaku, Tokyo, Japan). UV spectra were obtained in MeOH with a UV-2600 spectrometer (Shimadzu, Shanghai, China).

### 4.2. Optimization of Fermentation Medium for S. diacarni SCSIO 64983

In this experiment, 14 kinds of culture media were used for fermentation optimization. Spores were inoculated into a 250 mL Erlenmeyer flask containing 50 mL liquid culture medium and cultured in a shaker at 28 °C and 200 rpm. After 7 days, butanone was added for extraction, the crude extract was dissolved in methanol, the volume was adjusted to 500 μL, and samples were then analyzed by HPLC. The resulting data showed that N4 medium, out of all 14 possibilities, was optimal for *S. diacarni* SCSIO 64983. (The formulas of the 14 media used in this experiment and the HPLC analysis results for fermentation optimization are detailed in Supporting Information)

### 4.3. Scale-up Fermentation of S. diacarni SCSIO 64983

Strain SCSIO 64983 was activated on an ISP2 plate (yeast extract 4 g/L, glucose 4 g/L, Malt extract 10 g/L, sea salt 30 g/L, agar 20 g/L in purified water, pH 7.2–7.4) at 28 °C for 6 d. About 1 cm^2^ of spores and mycelium were scraped with bamboo sticks and inoculated into a 250 mL conical flask containing 50 mL N4 medium (pH: 7.2–7.4; starch 15 g/L, fish peptone 8 g/L, bacteriological peptone 5 g/L, glycerol 8 g/L, KBr 0.2 g/L, sea salt 30 g/L, and calcium carbonate 2 g/L), and were cultivated in a shaker at 28 °C 200 rpm for 2 days as seed solution. Subsequently, the seed solution was inoculated into a 1 L conical flask containing 200 mL of N4 medium, and was cultured in a shaker at 28 °C and 200 rpm for 7 days.

### 4.4. Extraction and Isolation of Compound **1**–**7**

*S. diacarni* SCSIO 64983 was fermented in N4 medium at 28 ℃, 200 rpm for 7 days. The fermentation broth (28 L) was centrifuged at a speed of 3900 rpm to separate the supernatant and mycelium; the mycelium was extracted with acetone, the supernatant was extracted with butanone, and the two extracts were then analyzed by HPLC. The two extracts were found to be similar and were accordingly combined. The combined extract was chromatographed on silica gel column with chloroform-methanol solvent (100:0, 98:2, 96:4, 94:6, 92:8, 9:1, 8:2, 7:3, 6: 4, 5:5, 0:100, *v/v*) gradient elution to afford fractions A1~A11 (Fr. A1~Fr. A11). Subsequent HPLC analysis of each fraction revealed that target compounds were concentrated in Fr. A1~Fr. A2. Accordingly, Fr. A1~Fr. A2 were combined and subjected to silica gel column chromatography using gradient elution with petroleum ether-ethyl acetate (95:5, 9:1, 8:2, 7:3, 6:4), 5:5, 3:7, 1:9, *v/v*); the resulting fractions were divided into Fr. B1~Fr. B10. Fr. B2 was eluted by HPLC semi-preparative column chromatography with MeCN-H_2_O (60:40) isocratic elution at a flow rate of 2.0 mL/min to afford compound **5** (*t*_R_ = 16.3 min, 72.0 mg). Fr. B1 was chromatographed on a semi-preparative HPLC column and eluted with MeCN-H_2_O (60:40) at a flow rate of 2.0 mL/min to render compound **4** (*t*_R_ = 20.3 min, 2.8 mg). Combined Fr. B5~Fr. B10, was then subjected to column chromatography, eluting with chloroform–methanol (50:50), to afford fractions Fr. C1~Fr. C15. TLC and HPLC analyses revealed that remaining target compounds were concentrated in fractions Fr. C4 and Fr. C12. Accordingly, Fr. C4 was subjected to semi-preparative HPLC purification using MeCN-H_2_O (55:45) as eluent to yield compound **3** (*t*_R_ = 22.0 min, 0.8 mg). Similarly, Fr. C12 was purified by semi-preparive HPLC [eluted with MeCN-H_2_O (40:60) at a flow rate of 2.0 mL/min] to render **2** (*t*_R_ = 20.0 min, 1 mg) and compound **1** (*t*_R_ = 23.6 min, 0.8 mg). Fr. A8 was eluted with a chloroform–methanol solvent gradient (100:0, 98:2, 96:4, 94:6, 92:8, 9:1, 8:2, 7:3, 6:4, 5: 5, 0:100, *v/v*) and fractions Fr. D1~Fr. D10 were obtained. HPLC analyses revealed that the remaining target compounds were concentrated in Fr. D2. Consequently, Fr. D2 was chromatographed using a semi-preparative HPLC column and elution with MeCN-H_2_O (60:40) at a flow rate of 2.0 mL/min to generate compound **7** (*t*_R_ = 11.0 min, 1.8 mg) and compound **6** (*t*_R_ = 9.0 min, 1.1 mg).

Thiocarbazomycin A (**1**), light brown powder; UV (MeOH) *λ*_max_ (log *ε*) 219 (4.46), 246 (4.32), 270 (4.33), and 291 (4.32) nm; HRESIMS date, see Figure S11. ^1^H and ^13^C NMR data, see Table 1 and Table S1.

Thiocarbazomycin B (**2**) was also isolated as a light brown solid; UV (MeOH) *λ*_max_ (log ε) 222 (4.03), 282 (3.83), 370 (3.08) nm; HRESIMS date, see Figure S23. ^1^H and ^13^C NMR data, see Table 1 and Table S1.

Chlocarbazomycin E (**3**), isolated as light brown amorphous powder; UV (MeOH) *λ*_max_ (log ε) 223 (3.86), 305 (3.54), 356 (2.85) nm; HRESIMS date, see Figure S31. ^1^H and ^13^C NMR data, see Table 1.

Brocarbazomycin A (**4**), was also isolated as a light brown solid. UV (MeOH) *λ*_max_ (log ε) 219 (3.85), 297 (3.33), and 344 (2.84) nm. HRESIMS date, see Figure S39. ^1^H and ^13^C NMR data, see Table 1.

### 4.5. Activity Test Method

The antibacterial activities of all compounds were assayed using the filter paper method: frozen glycerol stocks (−80 °C) of pathogenic bacteria were activated and transferred with LB liquid medium and then cultured at 37 °C, 200 rpm for 8–12 h; 100 μL of pathogenic bacteria liquid was spread with a sterile spreading rod and a sterile filter paper was placed in the center of each small square, respectively. At the center of each filter paper was placed 5 μL (10 μg) of test compound dissolved in DMSO, and then each plate was placed upside down in a 37 °C incubator for 16 h. Afterwards, zones of inhibited bacterial growth were observed and, if possible, measured.

## 5. Conclusions

In conclusion, we identified four microbial strains from coral *Acropora austera* and coral reef sand samples that can produce halocarbazomycins. Of the strains found, the most productive, *S. diacarni* SCSIO 64983, was fermented and found to generate seven halo- and thiocarbazomycins including chlocarbazomycins A–C (**5**–**7**) [13], thiocarbazomycins A–B (**1**–**2**), chlocarbazomycins E (**3**), and brocarbazomycin A (**4**). Isolation and characterization of the halo- and thiocarbazomycins revealed that compounds **1** and **2** share a 6/5/6/6 tetracyclic skeleton containing an S atom, whereas compound **4** has a rare aryl bromide group. The biological activities displayed by these compounds and their biosynthetic origins have not yet to be extensively explored but clearly warrant future efforts. 

## Figures and Tables

**Table 1:** ^1^H NMR (700 MHz) and ^13^C NMR (175 MHz) data of **1**–**4**.

Position	1^a^		2^a^		3^a^		4^b^	
	*δ*_C_, Type	*δ*_H_ (*J* in Hz)	*δ*_C_, Type	*δ*_H_ (*J* in Hz)	*δ*_C_, Type	*δ*_H_ (*J* in Hz)	*δ*_C_, Type	*δ*_H_ (*J* in Hz)
1	111.8, CH	7.45, br d (8.1)	110.7, CH	7.37, d (8.7)	108.9, CH	7.32, d (8.7)	109.6, CH	7.35, d (8.6)
2	126.8, CH	7.39, m	115.4, CH	7.27, d (8.7)	112.8, CH	7.20, d (8.7)	112.3, CH	7.11, d (8.6)
3	119.7, CH	7.17, m	150.8, C	-	148.3, C	-	150.2, C	-
4	123.6, CH	8.36, br d (7.9)	116.7, C	-	116.0, C	-	106.3, C	-
4a	123.8, C	-	122.3, C	-	120.9, C	-	123.4, C	-
4b	120.9, C	-	120.2, C	-	122.3, C	-	123.5, C	-
5	115.2, C	-	118.2, C	-	105.0, CH	8.04, d (2.4)	123.2, CH	8.83, br d (8.0)
6	130.6, C	-	130.9, C	-	153.0, C	-	119.3, CH	7.28, m
7	117.0, CH	7.07, d (8.5)	119.5, CH	7.14, d (8.6)	115.0, CH	7.08, dd (2.4, 8.8)	126.8, CH	7.47, m
8	110.1, CH	7.30, d (8.5)	110.6, CH	7.29, d (8.6)	110.9, CH	7.35, d (8.8)	110.6, CH	7.42, br d (8.0)
8a	138.2, C	-	139.7, C	-	136.2, C	-	140.7, C	-
9a	142.1, C	-	138.6, C	-	137.0, C	-	135.7, C	-
1‘’	167.1, C	-	167.0, C	-	-	-	-	-
2‘’	30.6, CH_2_	3.57, s	32.0, CH_2_	3.29, s	-	-	-	-
3-OMe	-	-	59.0, CH_3_	3.92, s	57.2, CH_3_	3.93, s	58.2, CH_3_	3.98, s
6-OMe	-	-	-	-	55.0, CH_3_	3.89, s	-	-

^a^ The data were recorded in methanol-*d*_4_; ^b^ the data were recorded in chloroform-*d*.

## Data Availability

The authors declare that all relevant data supporting the findings of this research can be found in the article and its supplementary materials or be obtained from the corresponding author upon request.

## Supplementary Materials

The following are available online at https://www.mdpi.com/article/10.3390/md20080537/s1, Figure S1–S61, Table S1: HRESIMS spectra, ^1^H (700 MHz) and ^13^C (175 MHz) NMR data for compound **1**–**7**. Figure S62: the HPLC analysis results for fermentation optimization of *S. diacarni* SCSIO 64983.
