# Supplementary material for: Halo- and Thiocarbazomycins from Coral- and Coral Reef Sands-Derived Actinomycetes
Source: Mar Drugs. 2022 Aug 21;20(8):537. doi: 10.3390/md20080537 (PMC9410401; doi:10.3390/md20080537)
Supplement: Supplementary file 1 [file marinedrugs-20-00537-s001.zip › marinedrugs-1871424-supplementary.pdf]

marine drugs

# Supporting information

## Halo- and Thiocarbazomycins from Coral- and Coral Reef Sands-Derived *Actinomycetes*

Qiaoling Wu [1,2], Hongjie Zhu[4], Changli Sun[1,3], Le Zhou[1]，Huimin Wang[5]，Songbiao Shi[1,2], Xinpeng Tian[1] and Jianhua Ju[1,2,3,*]

1 CAS Key Laboratory of Tropical Marine Bio-resources and Ecology, RNAM Center for Marine Microbiology, Guangdong Key Laboratory of Marine Materia Medica, South China Sea Institute of Oceanology, Chinese Academy of Sciences, Guangzhou, Guangdong 510301, China
2 University of Chinese Academy of Sciences, Beijing 110039, China
3 Southern Marine Science and Engineering Guangdong Laboratory, Guangzhou, Guangdong 511458, China
4 School of Pharmaceutical Sciences，Shandong University，Jinan 2500012，Shandong，China
5 School of Pharmacy, Institute of Marine Drug, Guangxi University of Traditional Chinese Medicine, Nanning 530200, China
* Correspondence: jju@scsio.ac.cn ; Tel.: +86-20-8902-3028

## Contents

1. NMR spectra of compounds 1-7. 3
Figure S1.$^1$H-NMR spectrum of compound 1 in mathanol-*d*4. 3
Figure S2.$^1$H-NMR spectrum of compound 1 in DMSO-*d*6. 4
Figure S3.$^{13}$C-NMR spectrum of compound 1 in mathanol-*d*4. 4
Figure S4.$^{13}$C-NMR spectrum of compound 1 in DMSO-*d*6. 5
Figure S5. DEPT135 spectrum of compound 1 in mathanol-*d*4. 5
Figure S6. HSQC spectrum of compound 1 in mathanol-*d*4. 6
Figure S7. HSQC spectrum of compound 1 in DMSO-*d*6. 6
Figure S8.$^1$H-$^1$H COSY spectrum of compound 1 in mathanol-*d*4. 7
Figure S9. HMBC spectrum of compound 1 in mathanol-*d*4. 7
Figure S10. HMBC spectrum of compound 1 in DMSO-*d*6. 7
Figure S11. HRESIMS of compound 1. 8
Figure S12. UV spectrum of compound 1. 9
Figure S13.$^1$H-NMR spectrum of compound 2 in mathanol-*d*4. 10
Figure S14.$^1$H-NMR spectrum of compound 2 in DMSO-*d*6. 10
Figure S15.$^{13}$C-NMR spectrum of compound 2 in mathanol-*d*4. 11
Figure S16.$^{13}$C-NMR spectrum of compound 2 in DMSO-*d*6. 11
Figure S17. DEPT135 spectrum of compound 2 in mathanol-*d*4. 12
Figure S18. HSQC spectrum of compound 2 in mathanol-*d*4. 12
Figure S19. HSQC spectrum of compound 2 in DMSO-*d*6. 13
Figure S20.$^1$H-$^1$H COSY spectrum of compound 2 in mathanol-*d*4. 13
Figure S21. HMBC spectrum of compound 2 in mathanol-*d*4. 14

Figure S22. HMBC spectrum of compound 2 in DMSO-$d_6$. 14
Figure S23. HRESIMS of compound 2. 15
Figure S24. UV spectrum of compound 2. 16
Figure S25. $^1$H-NMR spectrum of compound 3 in mathanol-$d_4$. 17
Figure S26. $^{13}$C-NMR spectrum of compound 3 in mathanol-$d_4$. 18
Figure S27. DEPT135 spectrum of compound 3 in mathanol-$d_4$. 18
Figure S28. HSQC spectrum of compound 3 in mathanol-$d_4$. 19
Figure S29. $^1$H-$^1$H COSY spectrum of compound 3 in mathanol-$d_4$. 19
Figure S30. HMBC spectrum of compound 3 in mathanol-$d_4$. 19
Figure S31. HRESIMS of compound 3. 20
Figure S32. UV spectrum of compound 3. 21
Figure S33. $^1$H-NMR spectrum of compound 4 in chloroform-*d*. 22
Figure S34. $^{13}$C-NMR spectrum of compound 4 in chloroform-*d*. 22
Figure S35. DEPT135 spectrum of compound 4 in chloroform-*d*. 23
Figure S36. HSQC spectrum of compound 4 in chloroform-*d*. 23
Figure S37. $^1$H-$^1$H COSY spectrum of compound 4 in chloroform-*d*. 24
Figure S38. HMBC spectrum of compound 4 in chloroform-*d*. 24
Figure S39. HRESIMS of compound 4. 25
Figure S40. UV spectrum of compound 4. 26
Figure S41. $^1$H-NMR spectrum of compound 5 in chloroform-*d*. 27
Figure S42. $^{13}$C-NMR spectrum of compound 5 in chloroform-*d*. 27
Figure S43. DEPT135 spectrum of compound 5 in chloroform-*d*. 28
Figure S44. HSQC spectrum of compound 5 in chloroform-*d*. 28
Figure S45. $^1$H-$^1$H COSY spectrum of compound 5 in chloroform-*d*. 29
Figure S46. HMBC spectrum of compound 5 in chloroform-*d*. 29
Figure S47. HRESIMS of compound 5. 30
Figure S48. $^1$H-NMR spectrum of compound 6 in mathanol-$d_4$. 30
Figure S49. $^{13}$C-NMR spectrum of compound 6 in mathanol-$d_4$. 31
Figure S50. DEPT135 spectrum of compound 6 in mathanol-$d_4$. 31
Figure S51. HSQC spectrum of compound 6 in mathanol-$d_4$. 32
Figure S52. $^1$H-$^1$H COSY spectrum of compound 6 in mathanol-$d_4$. 32
Figure S53. HMBC spectrum of compound 6 in mathanol-$d_4$. 33
Figure S54. HRESIMS of compound 6. 33
Figure S55. $^1$H-NMR spectrum of compound 7 in mathanol-$d_4$. 34
Figure S56. $^{13}$C-NMR spectrum of compound 7 in mathanol-$d_4$. 34
Figure S57. DEPT135 spectrum of compound 7 in mathanol-$d_4$. 35
Figure S58. HSQC spectrum of compound 7 in mathanol-$d_4$. 35
Figure S59. $^1$H-$^1$H COSY spectrum of compound 7 in mathanol-$d_4$. 36
Figure S60. HMBC spectrum of compound 7 in mathanol-$d_4$. 36
Figure S61. HRESIMS of compound 7. 37
2. Medium Formula and Optimization Results of *S. diacarni* SCSIO 64983 37
Figure S62. Optimization Results of *S. diacarni* SCSIO 64983 38

3. NMR data of 6-7 in methanol-$d_4$ and 1–2 in DMSO- $d_6$ 38

# **1.** NMR spectra of compounds **1-7.**

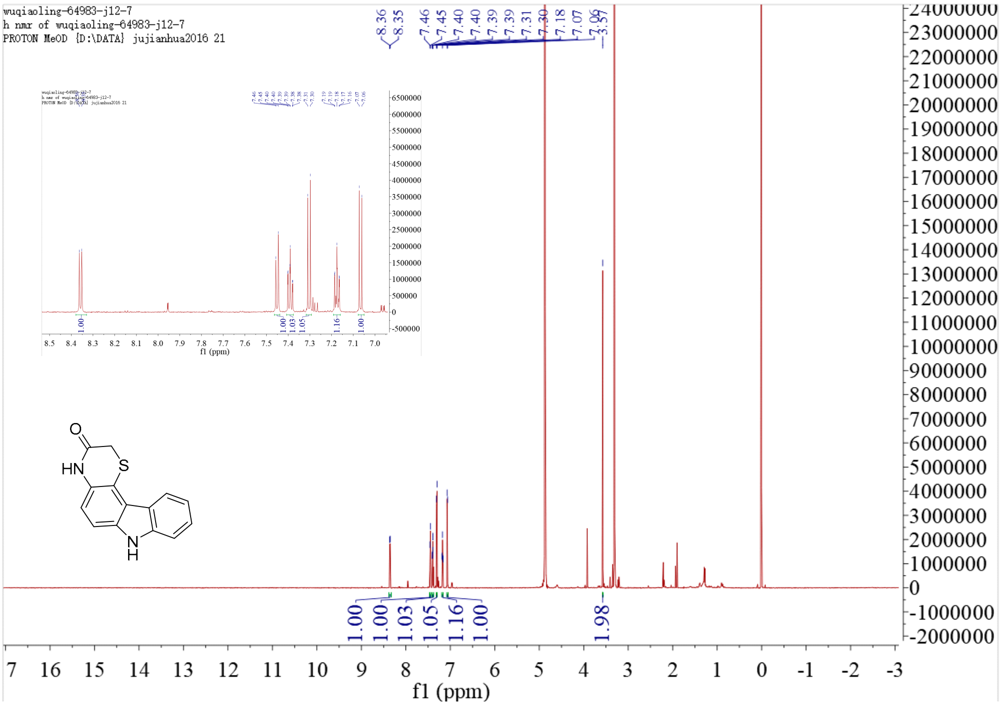


**Figure S1.** $^{1}$H-NMR spectrum of compound **1** in mathanol-$d_4$.

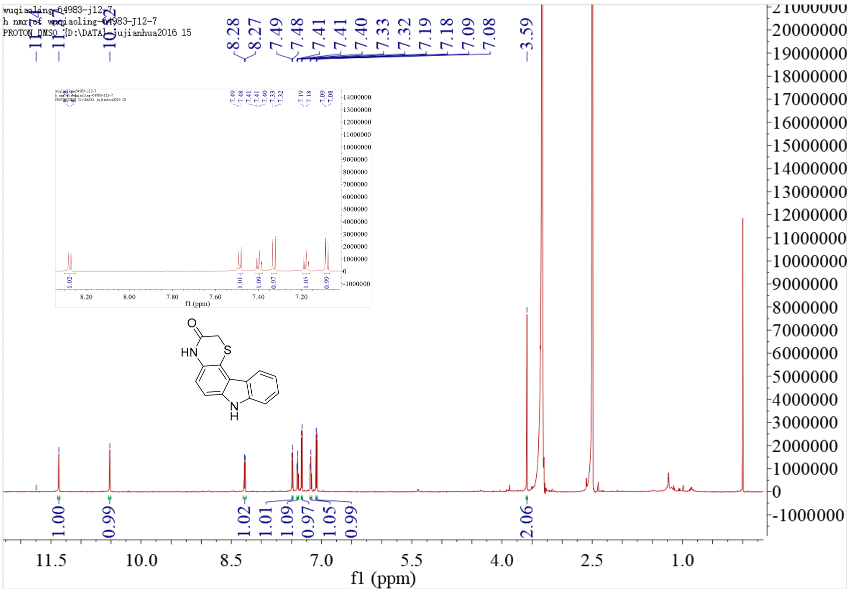

**Figure S2.** $^{1}$H-NMR spectrum of compound **1** in DMSO-$d_6$.

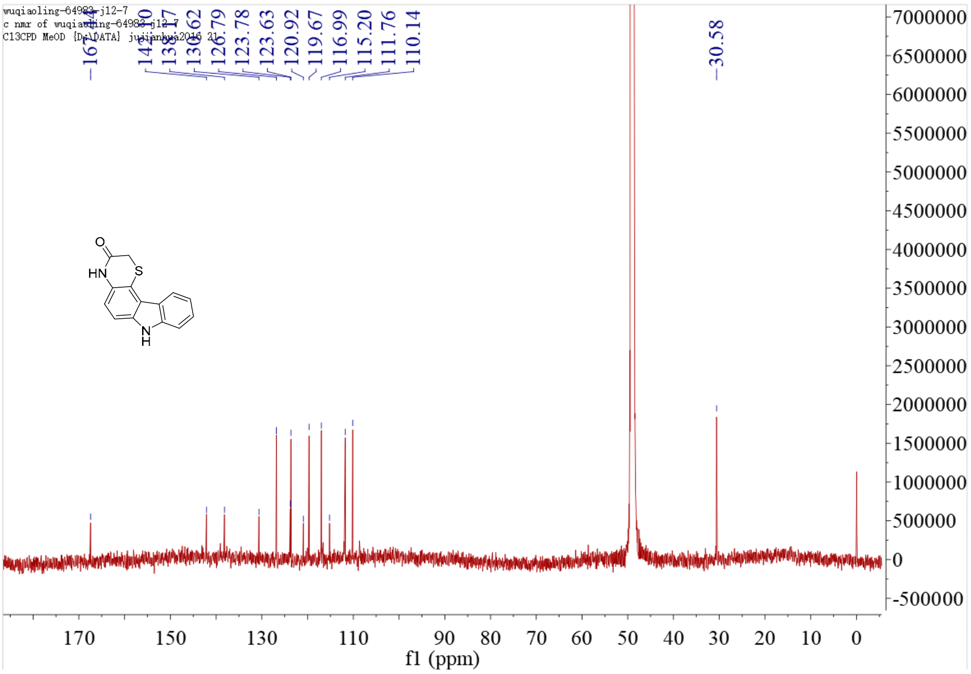


**Figure S3.** $^{13}$C-NMR spectrum of compound **1** in mathanol-$d_4$.

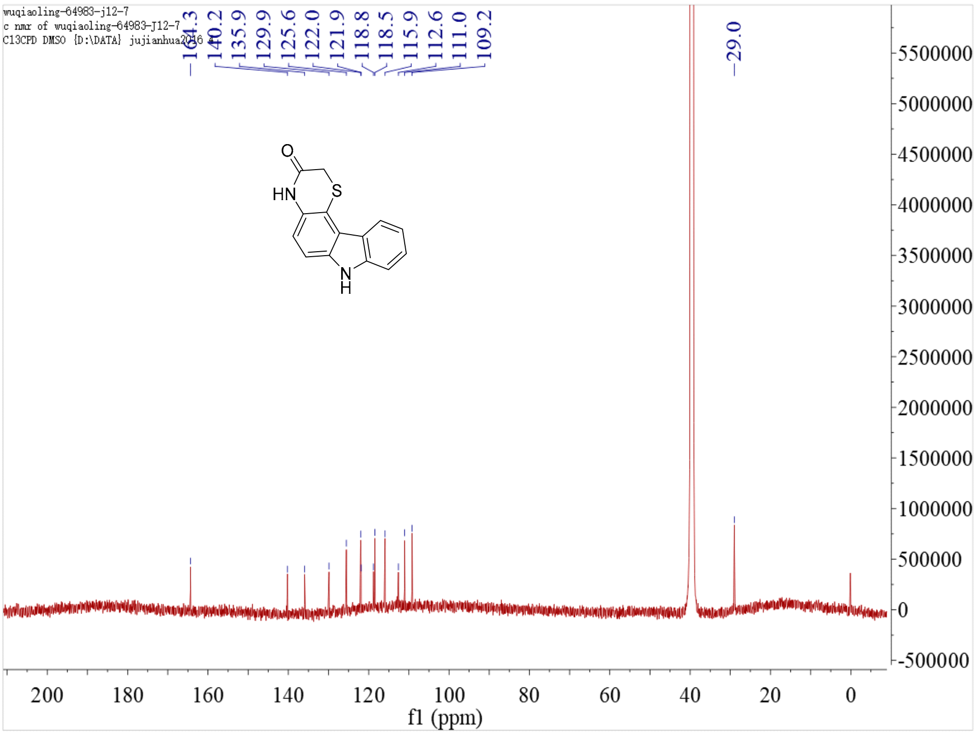

**Figure S4.** $^{13}$C-NMR spectrum of compound **1** in DMSO-*d*6.

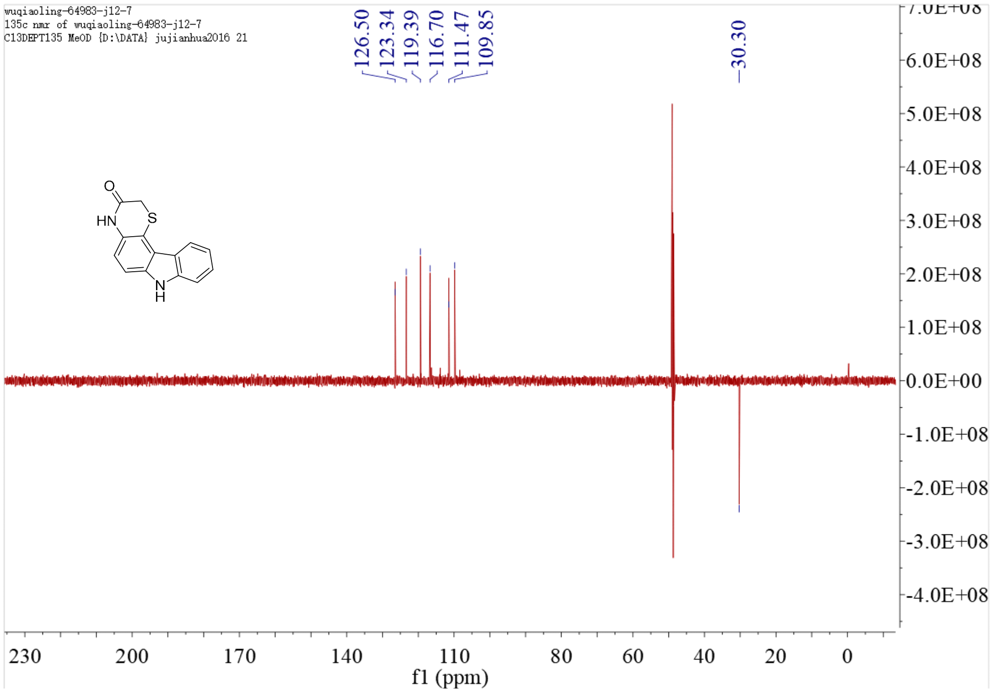


**Figure S5**. DEPT135 spectrum of compound **1** in mathanol-*d*4.

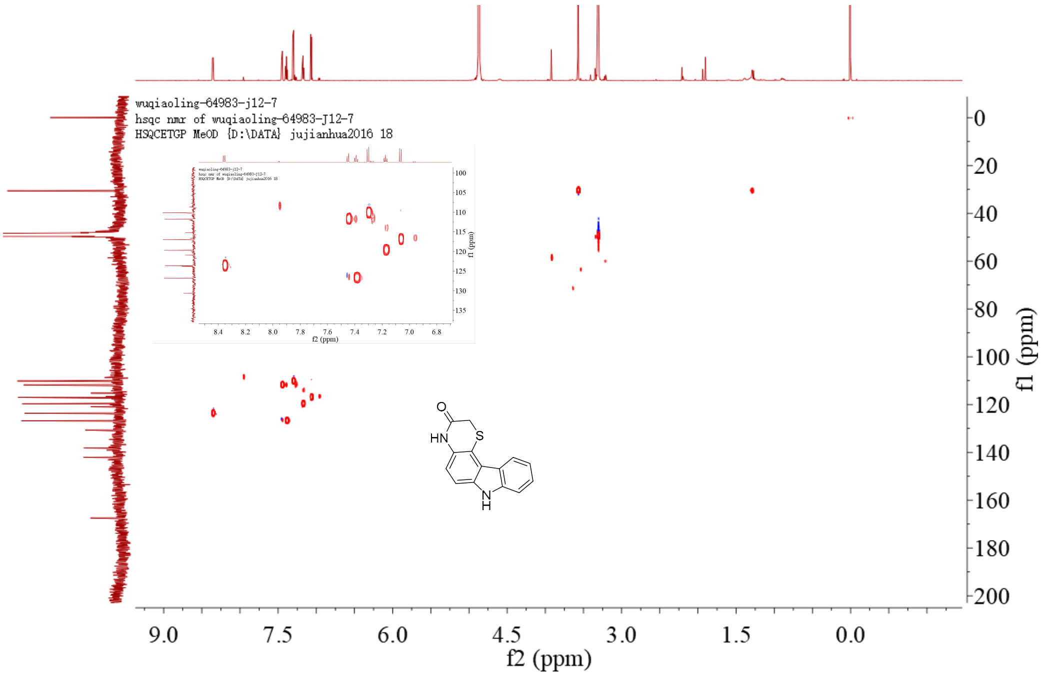

**Figure S6**. HSQC spectrum of compound **1** in mathanol-*d*4.

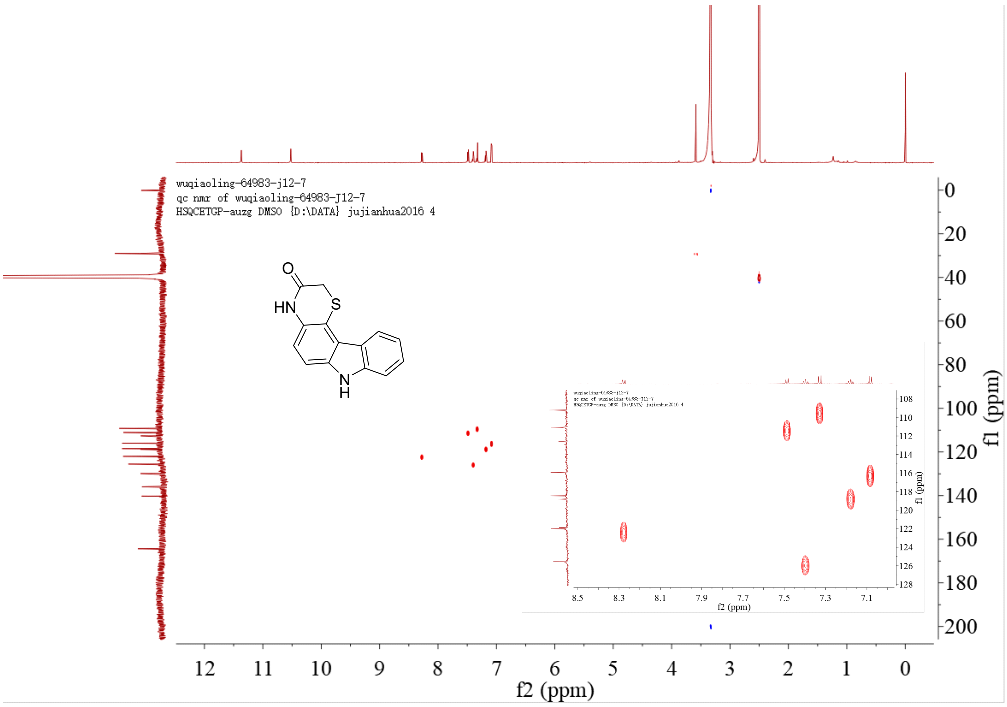


**Figure S7**. HSQC spectrum of compound **1** in DMSO-*d*6.

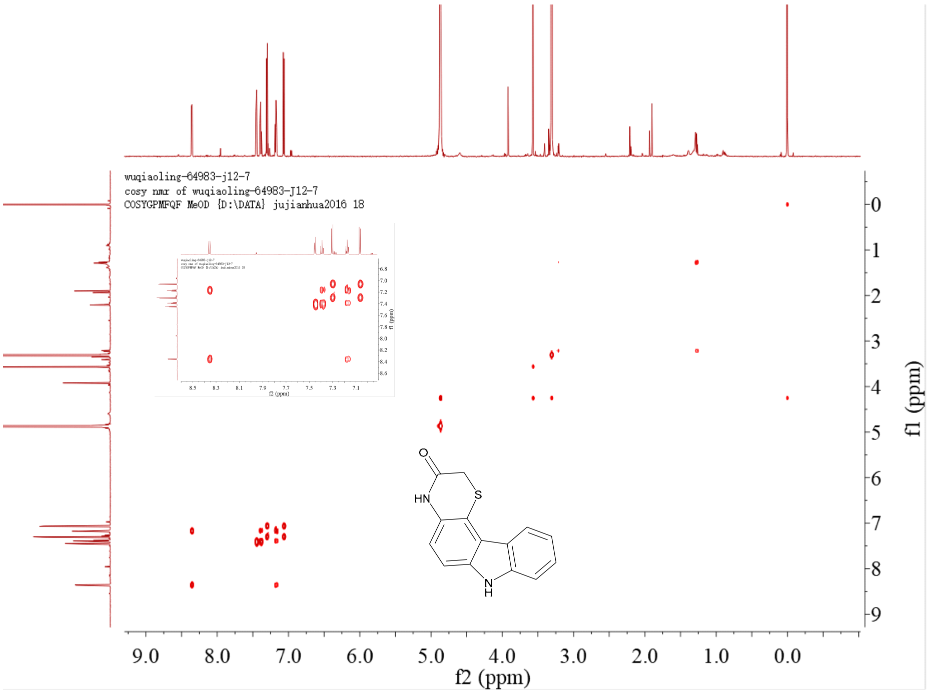

**Figure S8.** $^{1}H$-$^{1}H$ COSY spectrum of compound **1** in mathanol-*d*4.

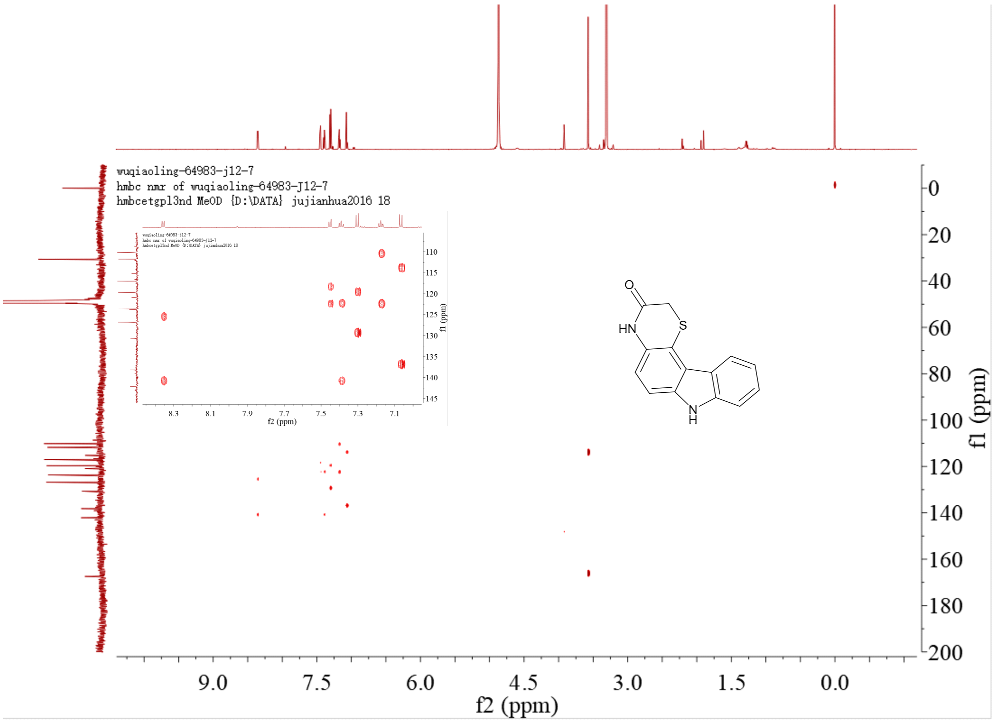


**Figure S9.** HMBC spectrum of compound **1** in mathanol-*d*4.

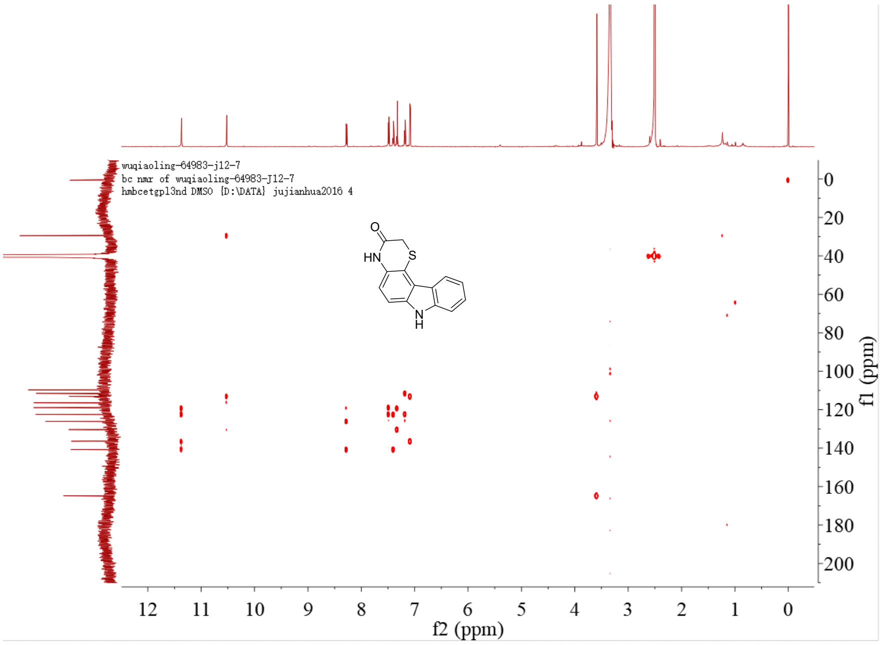


**Figure S10**. HMBC spectrum of compound **1** in DMSO-*d*6.

## Mass Spectrum SmartFormula Report

**Analysis Info**

| | | | |
|---|---|---|---|
| Analysis Name | D:\Data\MS\data\202102\wuqiaoling_64983-J12-7_pos_72_01_10009.d | Acquisition Date | 2/8/2021 5:08:44 PM |
| Method | LC_Direct Infusion_pos_70-500mz.m | Operator | SCSIO |
| Sample Name | wuqiaoling_64983-J12-7_pos | Instrument | maXis 255552.00029 |
| Comment | | | |

**Acquisition Parameter**

| | | | | | |
|---|---|---|---|---|---|
| Source Type | ESI | Ion Polarity | Positive | Set Nebulizer | 0.4 Bar |
| Focus | Active | Set Capillary | 4500 V | Set Dry Heater | 180 °C |
| Scan Begin | 70 m/z | Set End Plate Offset | -500 V | Set Dry Gas | 4.0 l/min |
| Scan End | 1500 m/z | Set Charging Voltage | 0 V | Set Divert Valve | Waste |
| | | Set Corona | 0 nA | Set APCI Heater | 0 °C |

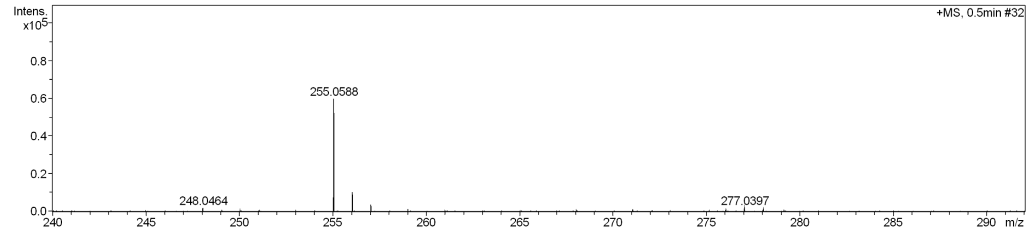


| Meas. m/z | # | Ion Formula | Score | m/z | err [ppm] | err [mDa] | mSigma | rdb | e⁻ Conf | N-Rule |
|---|---|---|---|---|---|---|---|---|---|---|
| 255.0588 | 1 | C14H11N2OS | 100.00 | 255.0587 | -0.5 | -0.1 | 1.3 | 10.5 | even | ok |
| 277.0397 | 1 | C14H10N2NaOS | 100.00 | 277.0406 | 3.4 | 0.9 | 111.1 | 10.5 | even | ok |

wuqiaoling_64983-J12-7_pos_72_01_10009.d
Bruker Compass DataAnalysis 4.1 printed: 7/28/2022 5:39:59 PM by: SCSIO Page 1 of 1

**Figure S11**. HRESIMS of compound **1**.

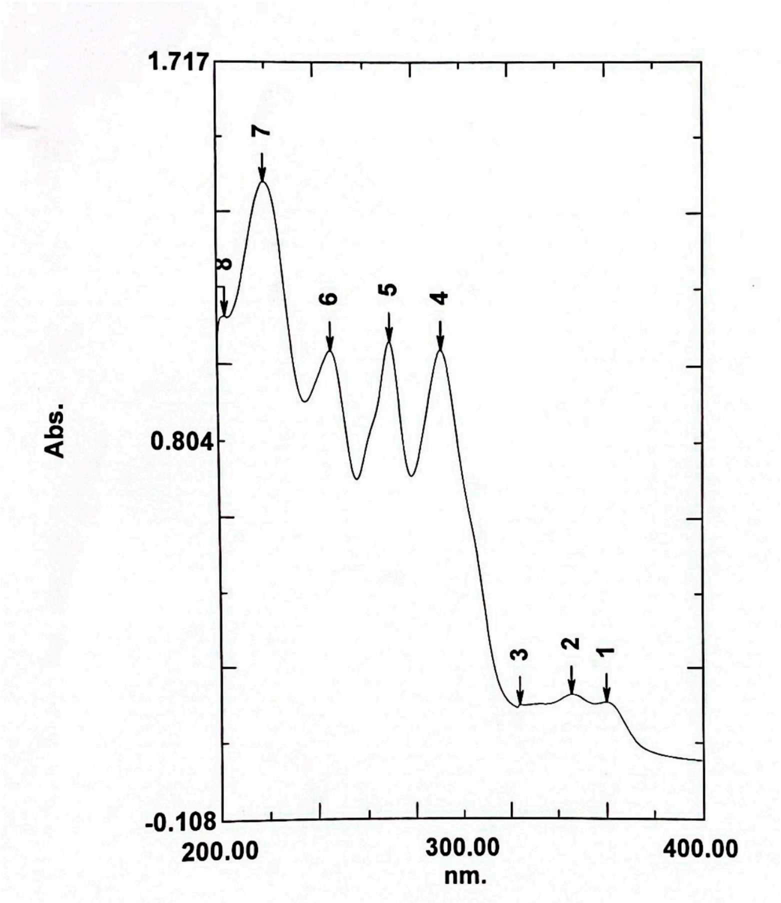


**Figure S12**. UV spectrum of compound **1**.

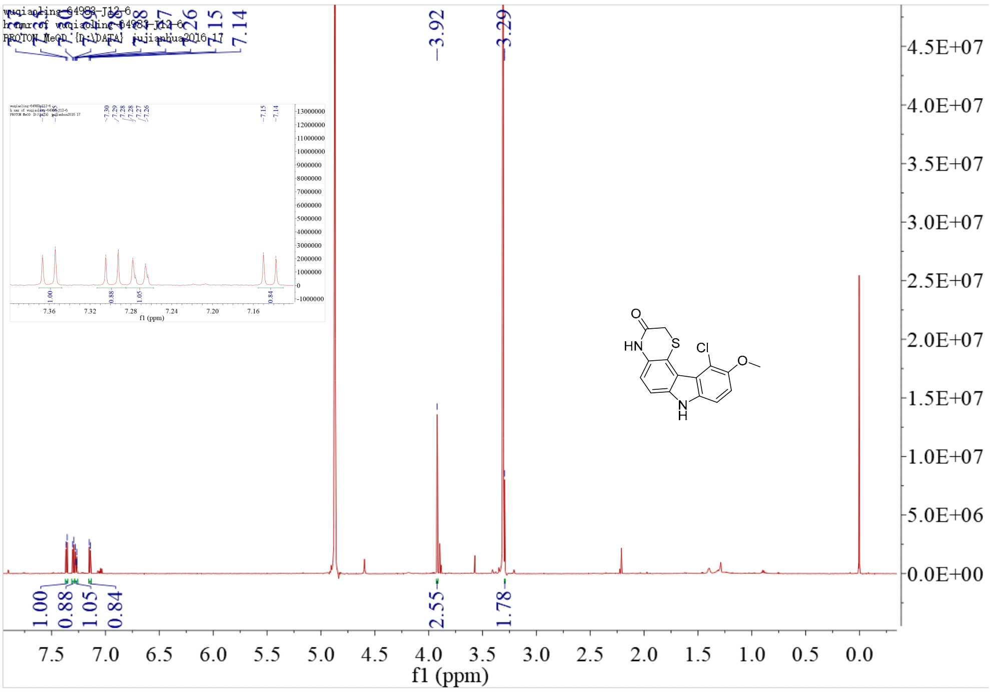

**Figure S13**.$^{1}$H-NMR spectrum of compound **2** in mathanol-*d*4.

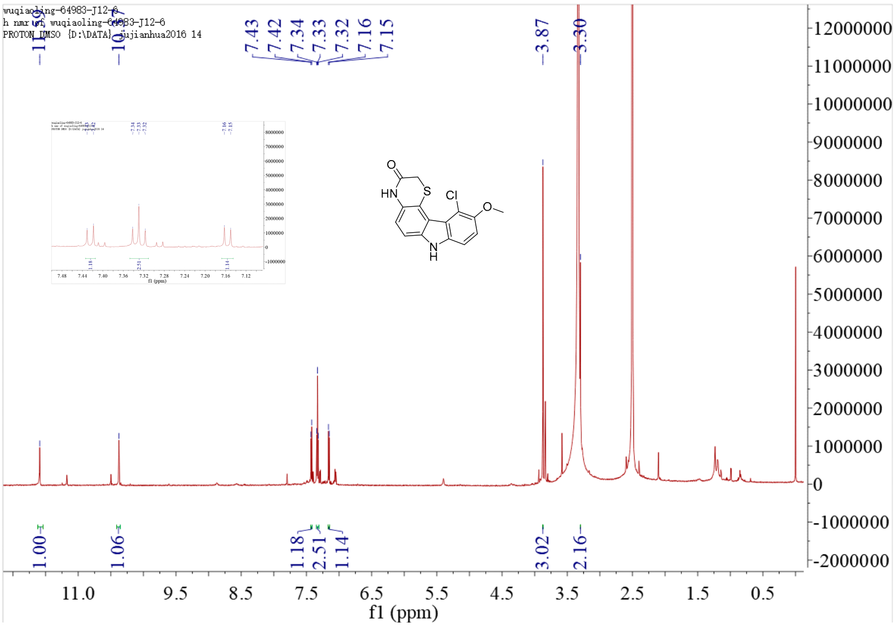

**Figure S14**.$^{1}$H-NMR spectrum of compound **2** in DMSO-*d*6.

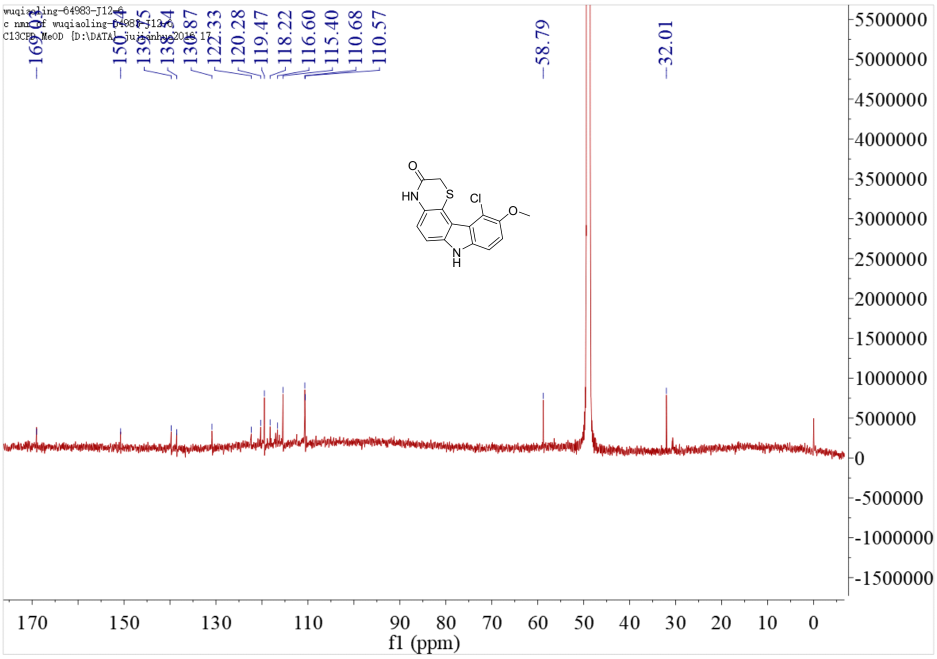

**Figure S15.** $^{13}$C-NMR spectrum of compound **2** in mathanol-*d*4.

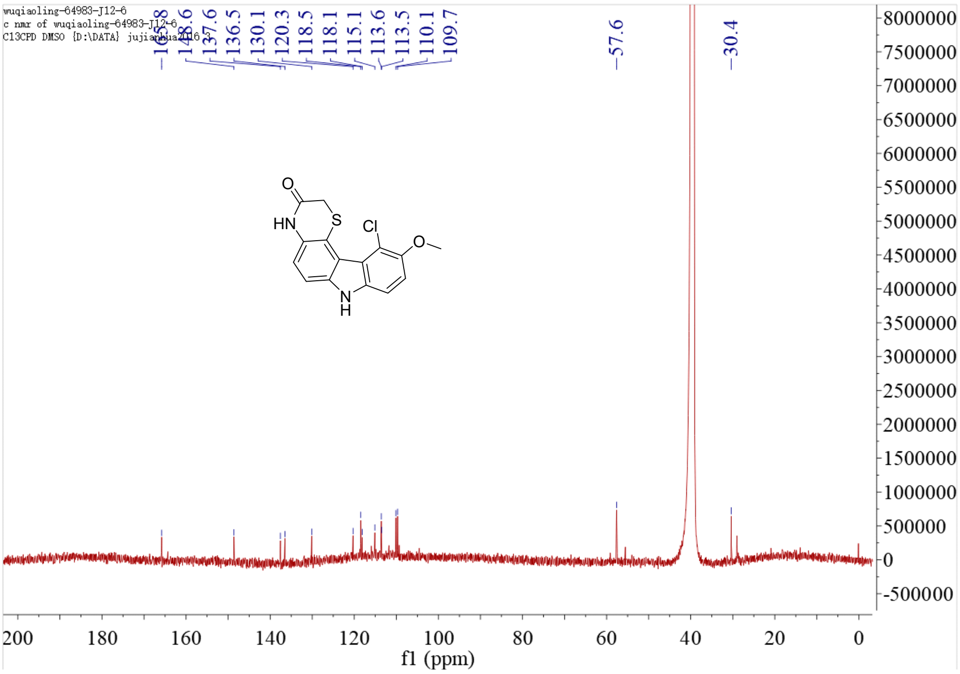

**Figure S16.** $^{13}$C-NMR spectrum of compound **2** in DMSO-*d*6.

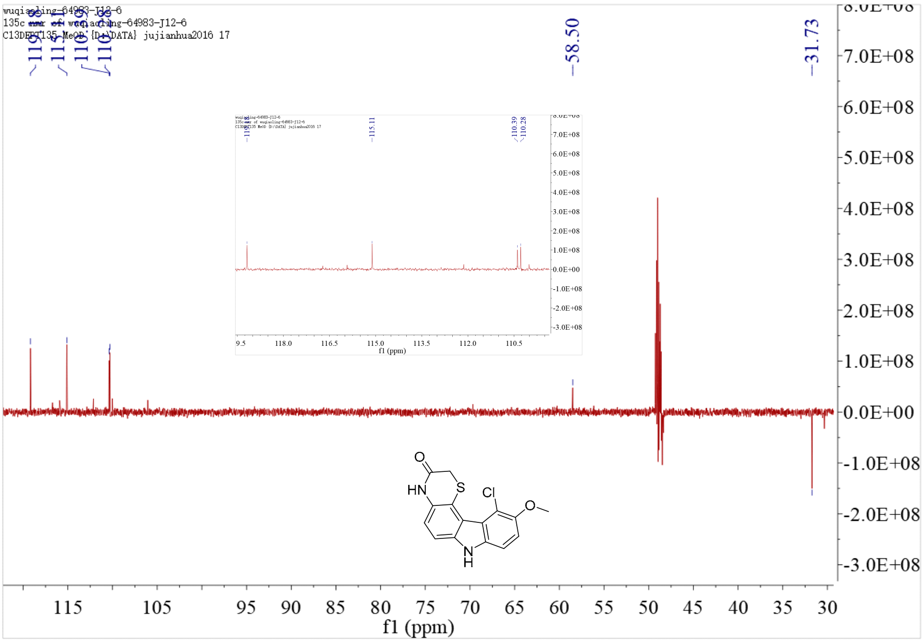


**Figure S17**. DEPT135 spectrum of compound **2** in mathanol-*d*4.

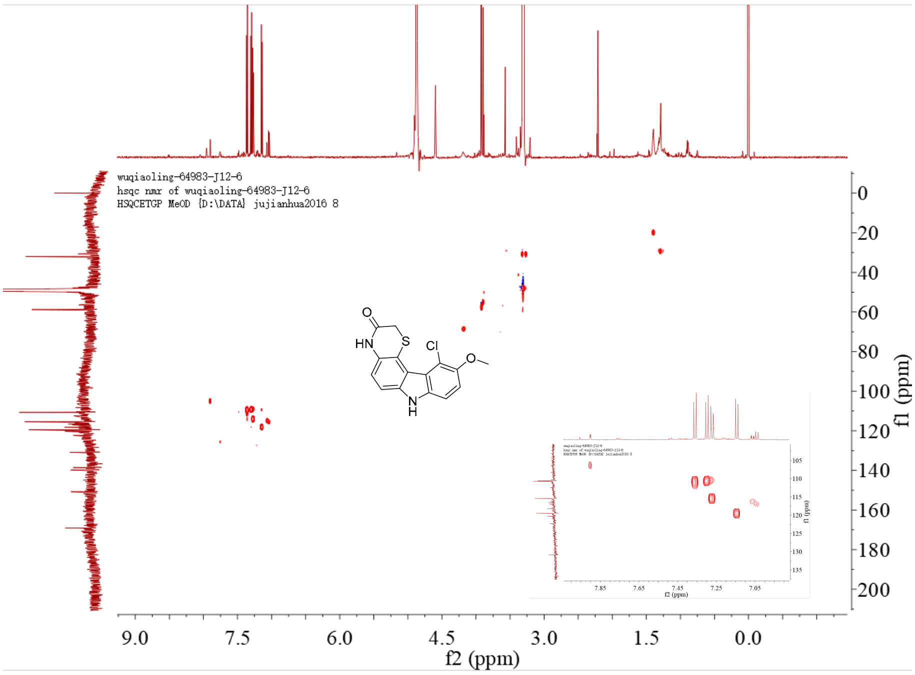


**Figure S18**. HSQC spectrum of compound **2** in mathanol-*d*4.

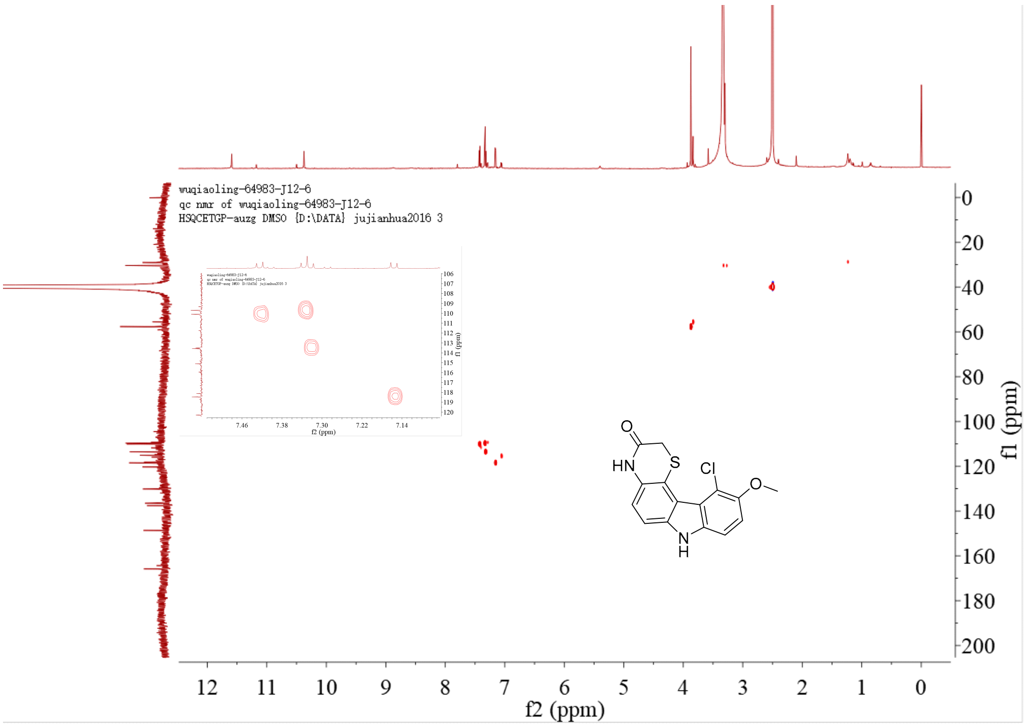


**Figure S19**. HSQC spectrum of compound **2** in DMSO-$d_6$.

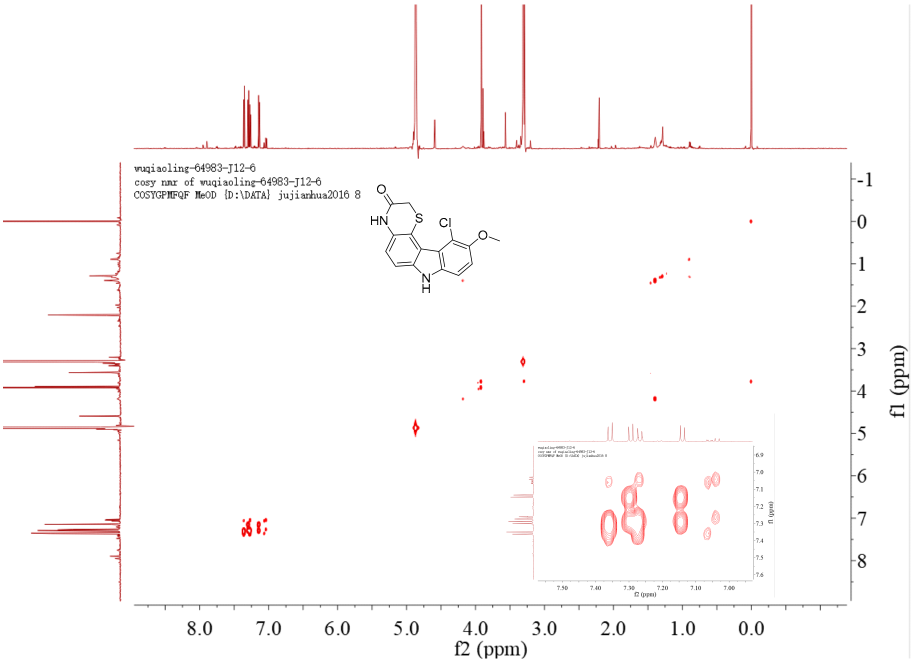


**Figure S20**. $^1$H-$^1$H COSY spectrum of compound **2** in mathanol-$d_4$.

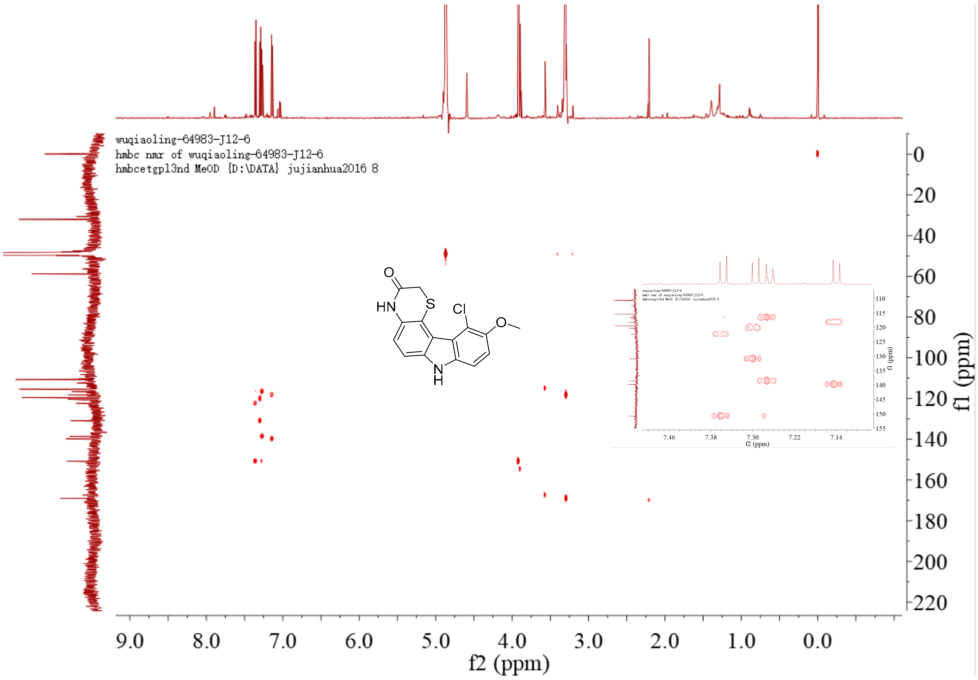


**Figure S21**. HMBC spectrum of compound **2** in mathanol-$d_4$.

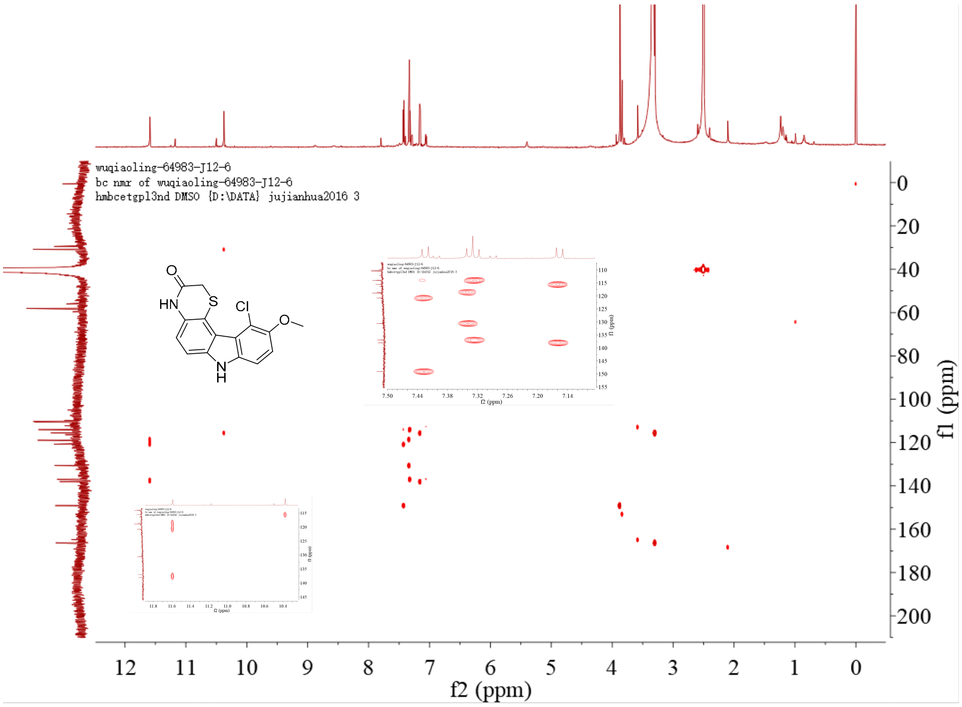


**Figure S22**. HMBC spectrum of compound **2** in DMSO-$d_6$.

## Mass Spectrum SmartFormula Report

**Analysis Info**

| | | | |
|---|---|---|---|
| Analysis Name | D:\Data\MS\data\202102\wuqiaoling_64983-J12-6_pos_71_01_10008.d | Acquisition Date | 2/8/2021 5:05:24 PM |
| Method | LC_Direct Infusion_pos_70-500mz.m | Operator | SCSIO |
| Sample Name | wuqiaoling_64983-J12-6_pos | Instrument | maXis 255552.00029 |
| Comment | | | |

**Acquisition Parameter**

| | | | | | |
|---|---|---|---|---|---|
| Source Type | ESI | Ion Polarity | Positive | Set Nebulizer | 0.4 Bar |
| Focus | Active | Set Capillary | 4500 V | Set Dry Heater | 180 °C |
| Scan Begin | 70 m/z | Set End Plate Offset | -500 V | Set Dry Gas | 4.0 l/min |
| Scan End | 1500 m/z | Set Charging Voltage | 0 V | Set Divert Valve | Waste |
| | | Set Corona | 0 nA | Set APCI Heater | 0 °C |

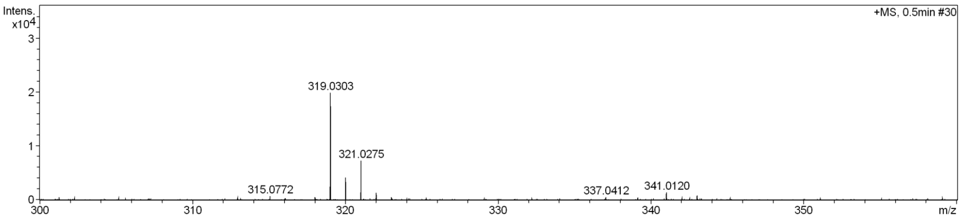


| Meas. m/z | # | Ion Formula | Score | m/z | err [ppm] | err [mDa] | mSigma | rdb | e⁻ Conf | N-Rule |
|---|---|---|---|---|---|---|---|---|---|---|
| 319.0303 | 1 | C15H12ClN2O2S | 100.00 | 319.0303 | 0.2 | 0.1 | 14.3 | 10.5 | even | ok |
| 341.0120 | 1 | C15H11ClN2NaO2S | 100.00 | 341.0122 | 0.7 | 0.2 | 88.8 | 10.5 | even | ok |

wuqiaoling_64983-J12-6_pos_71_01_10008.d
Bruker Compass DataAnalysis 4.1 printed: 7/28/2022 5:39:27 PM by: SCSIO Page 1 of 1

**Figure S23**. HRESIMS of compound **2**.

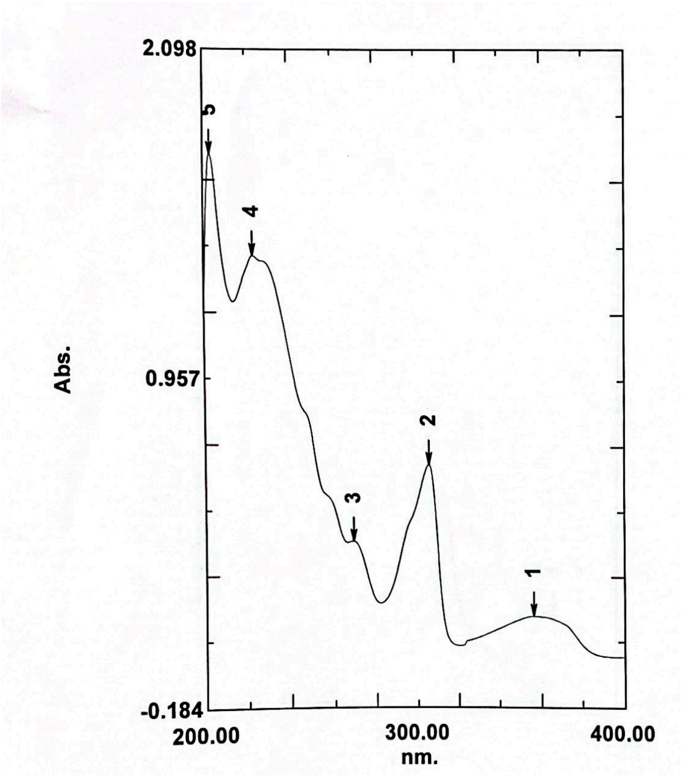


**Figure S24**. UV spectrum of compound **2**.

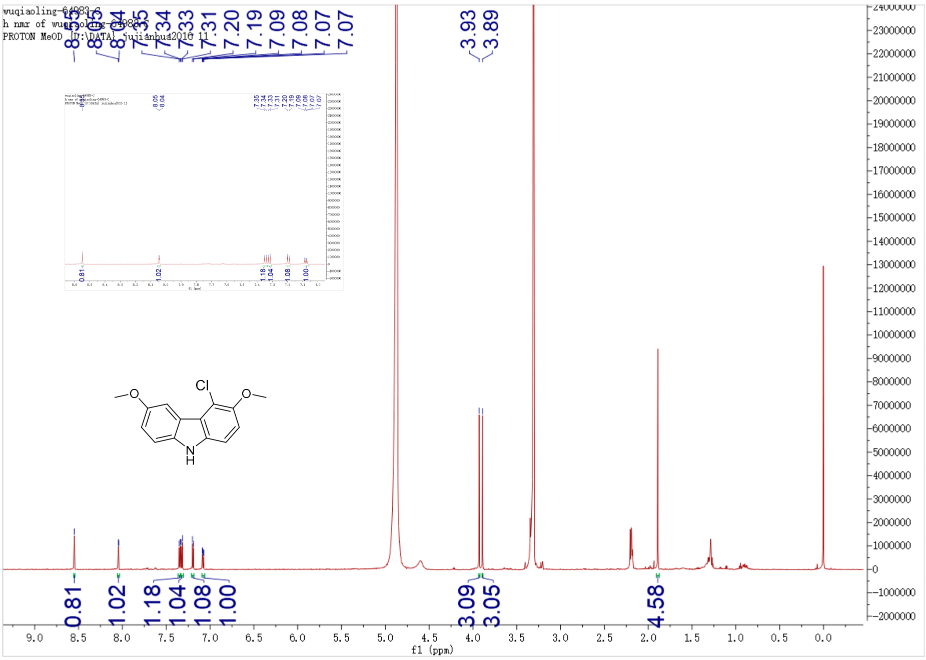


**Figure S25**.$^{1}$H-NMR spectrum of compound **3** in mathanol-*d*4.

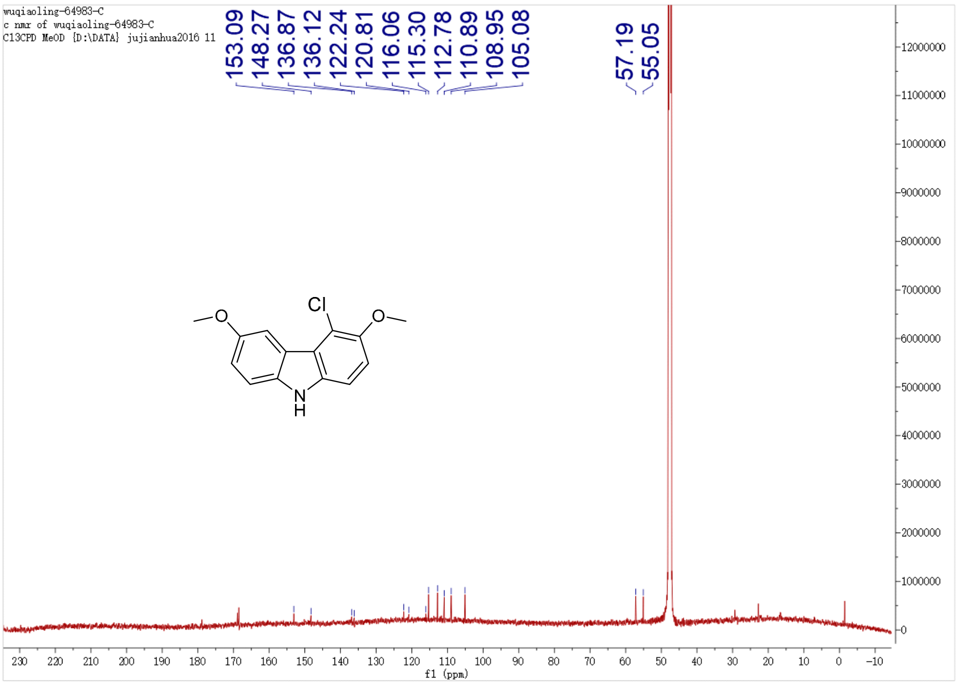

**Figure S26.** $^{13}$C-NMR spectrum of compound **3** in mathanol-$d_4$.

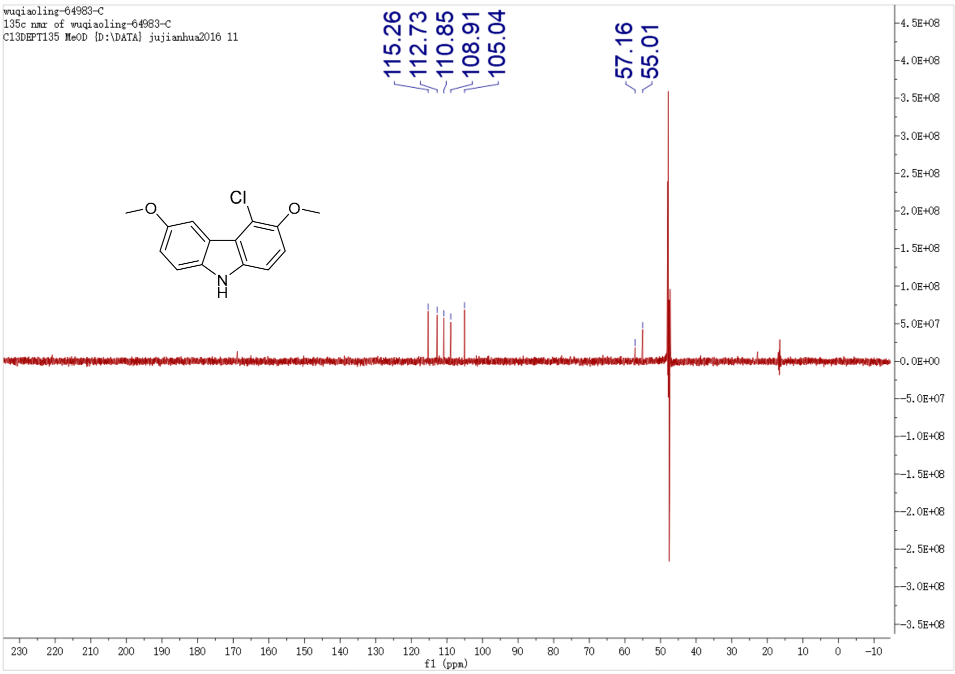


**Figure S27**. DEPT135 spectrum of compound **3** in mathanol-$d_4$.

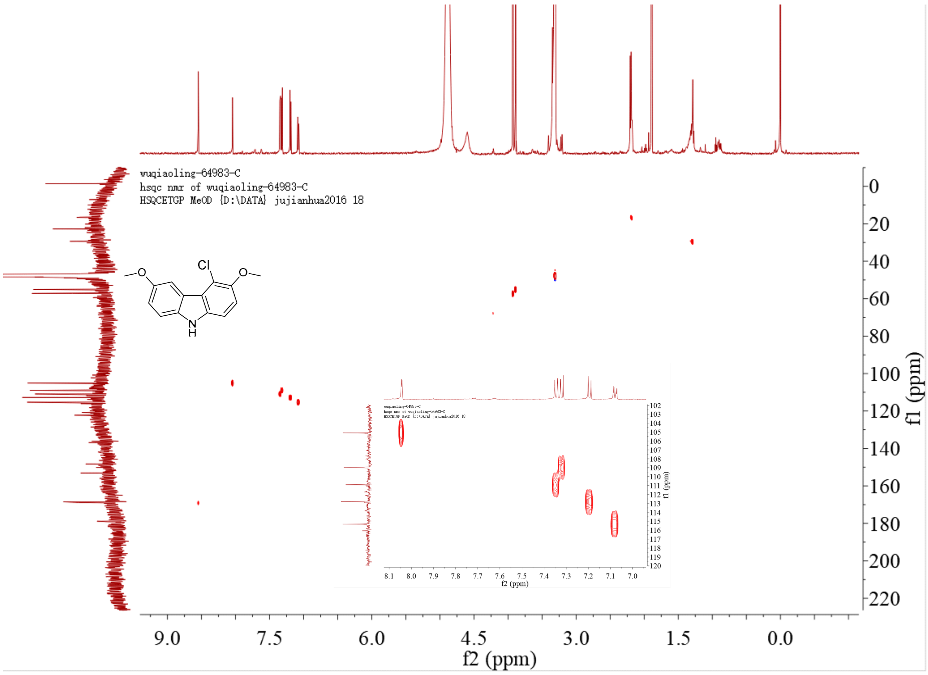

**Figure S28**. HSQC spectrum of compound **3** in mathanol-*d*4.

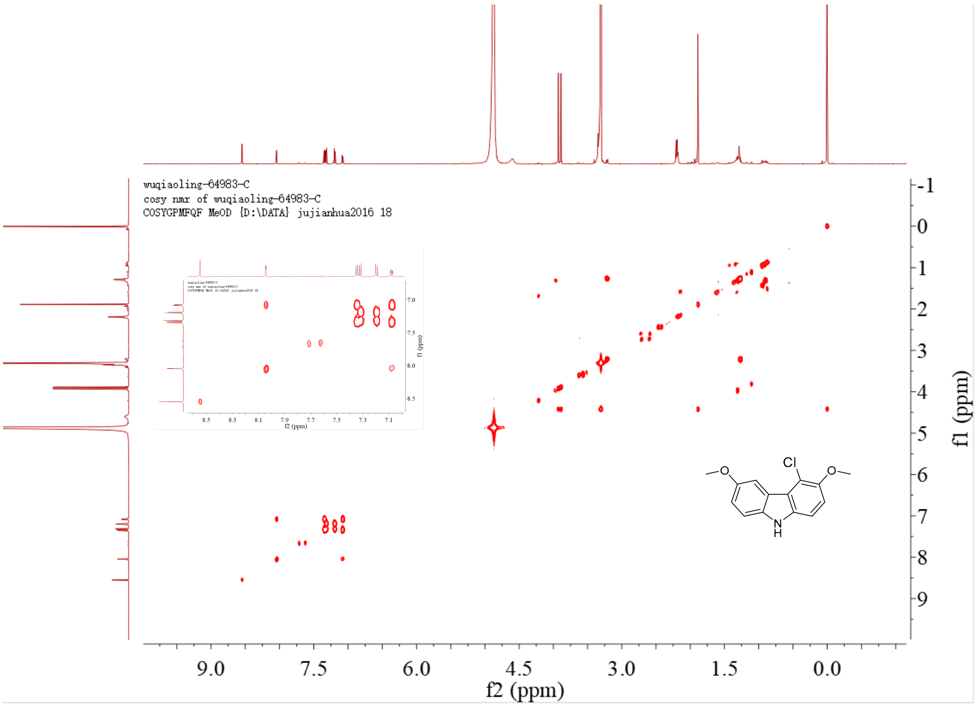


**Figure S29.$^{1}$H-$^{1}$H COSY spectrum of compound 3 in mathanol-*d*4.**

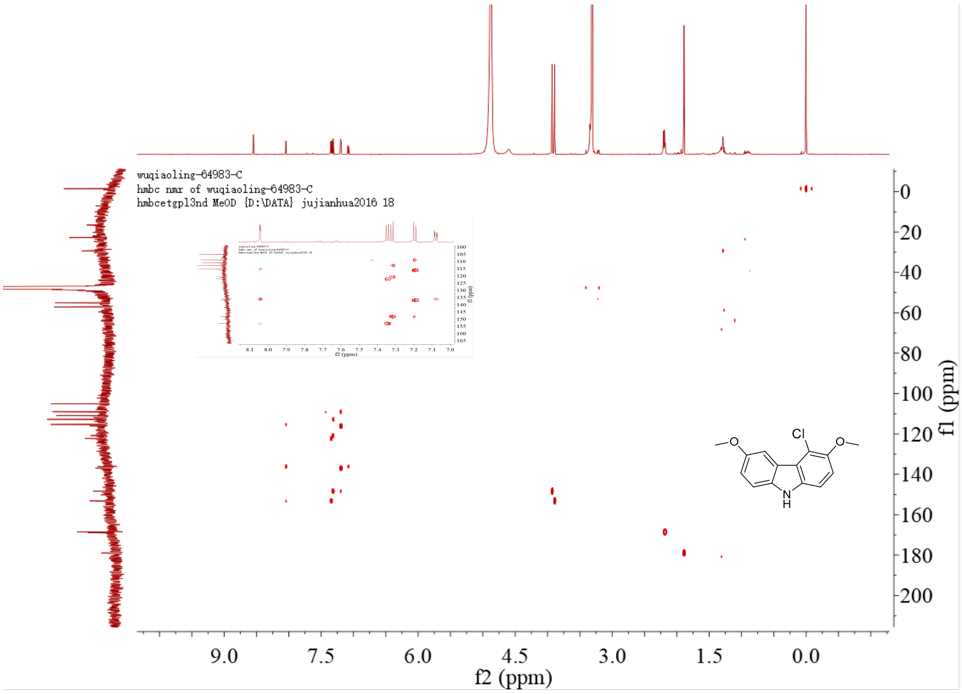


**Figure S30. HMBC spectrum of compound 3 in mathanol-*d*4.**

## Mass Spectrum SmartFormula Report

**Analysis Info**

| | | | |
|---|---|---|---|
| Analysis Name | D:\Data\MS\data\202104\wuqiaoling_64983-C_pos_71_01_10589.d | Acquisition Date | 4/29/2021 5:45:34 PM |
| Method | LC_Direct Infusion_pos_70-500mz.m | Operator | SCSIO |
| Sample Name | wuqiaoling_64983-C_pos | Instrument | maXis 255552.00029 |
| Comment | | | |

**Acquisition Parameter**

| | | | | | |
|---|---|---|---|---|---|
| Source Type | ESI | Ion Polarity | Positive | Set Nebulizer | 0.4 Bar |
| Focus | Active | Set Capillary | 4500 V | Set Dry Heater | 180 °C |
| Scan Begin | 70 m/z | Set End Plate Offset | -500 V | Set Dry Gas | 4.0 l/min |
| Scan End | 1500 m/z | Set Charging Voltage | 0 V | Set Divert Valve | Waste |
| | | Set Corona | 0 nA | Set APCI Heater | 0 °C |

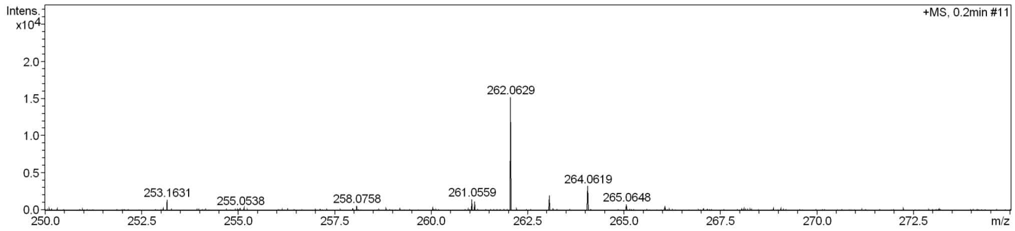


| Meas. m/z | # | Ion Formula | Score | m/z | err [ppm] | err [mDa] | mSigma | rdb | e⁻ Conf | N-Rule |
|---|---|---|---|---|---|---|---|---|---|---|
| 262.0629 | 1 | C14H13ClNO2 | 100.00 | 262.0629 | 0.3 | 0.1 | 56.6 | 8.5 | even | ok |

wuqiaoling_64983-C_pos_71_01_10589.d
Bruker Compass DataAnalysis 4.1 printed: 8/1/2022 4:24:59 PM by: SCSIO Page 1 of 1

**Figure S31.** HRESIMS of compound **3**.

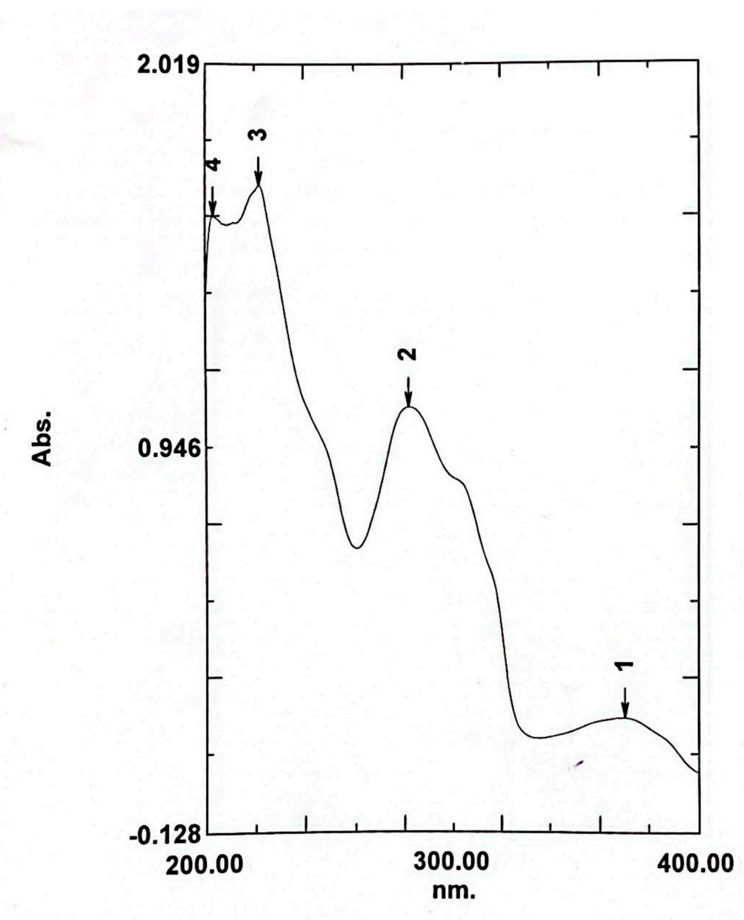


**Figure S32**. UV spectrum of compound **3**.

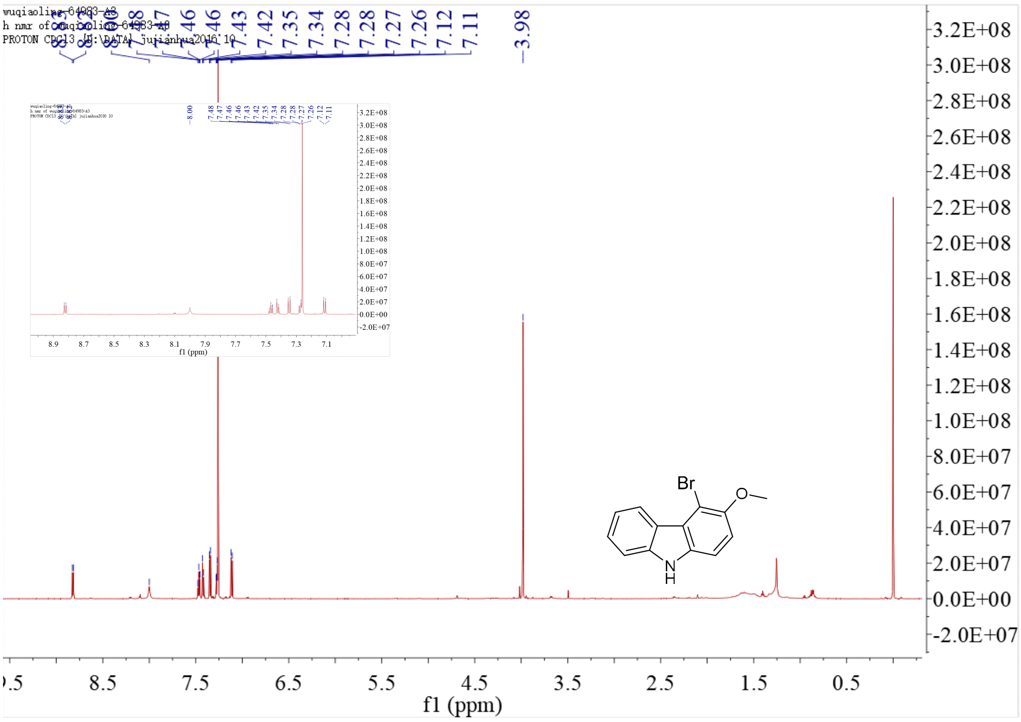


**Figure S33**.$^{1}$H-NMR spectrum of compound **4** in chloroform-*d*.

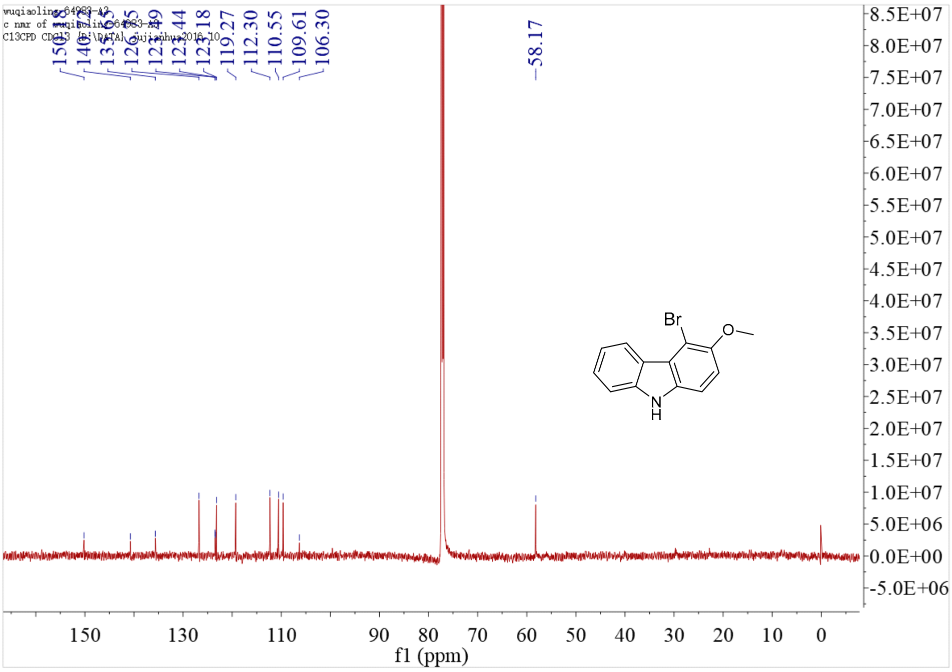


**Figure S34**.$^{13}$C-NMR spectrum of compound **4** in chloroform-*d*.

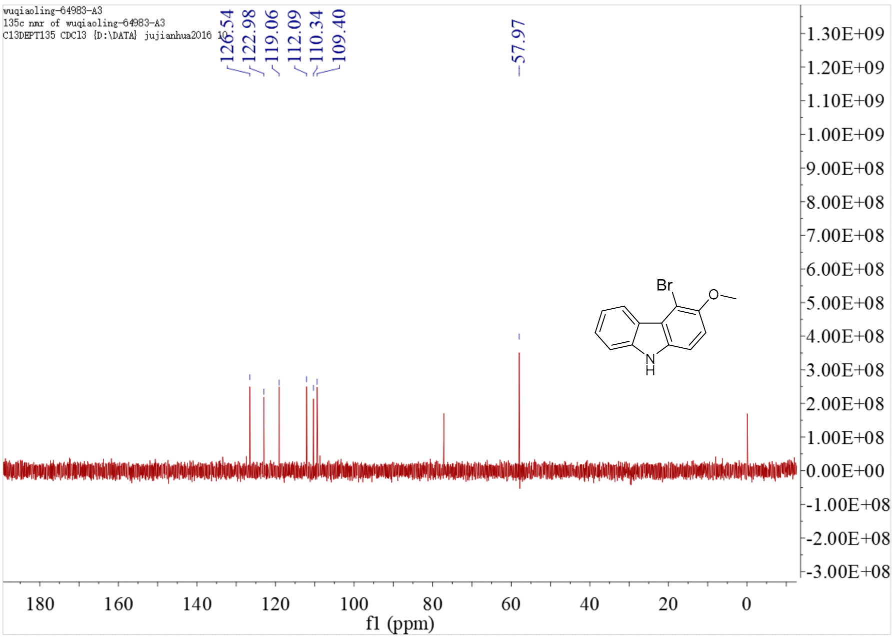


**Figure S35**. DEPT135 spectrum of compound **4** in chloroform-*d*.

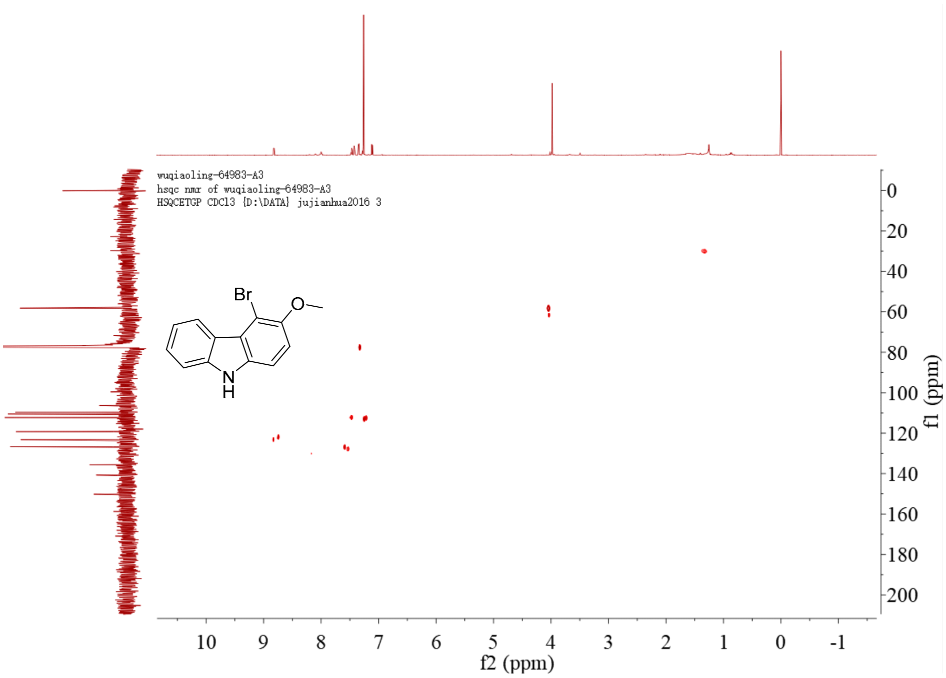


**Figure S36.** HSQC spectrum of compound **4** in chloroform-*d*.

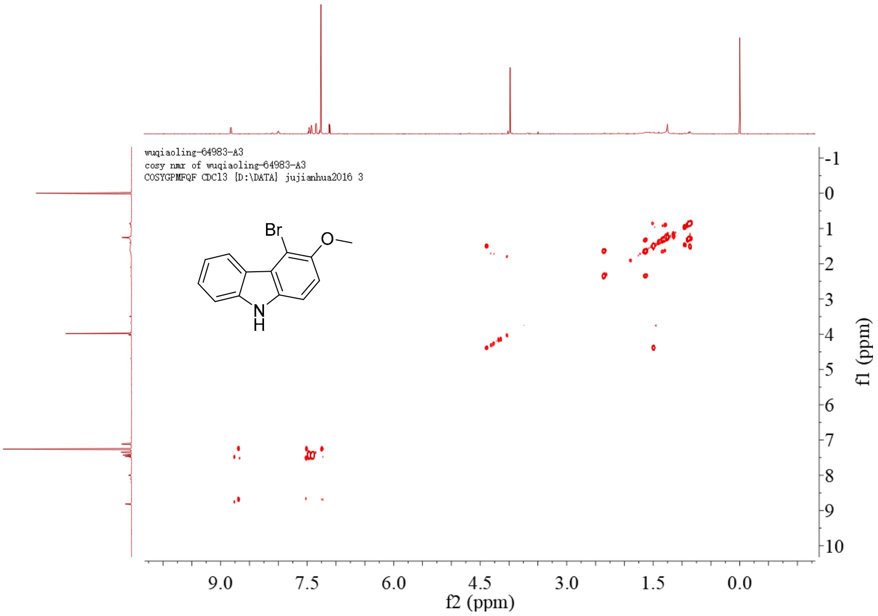


**Figure S37.** $^{1}$H-$^{1}$H COSY spectrum of compound **4** in chloroform-*d*.

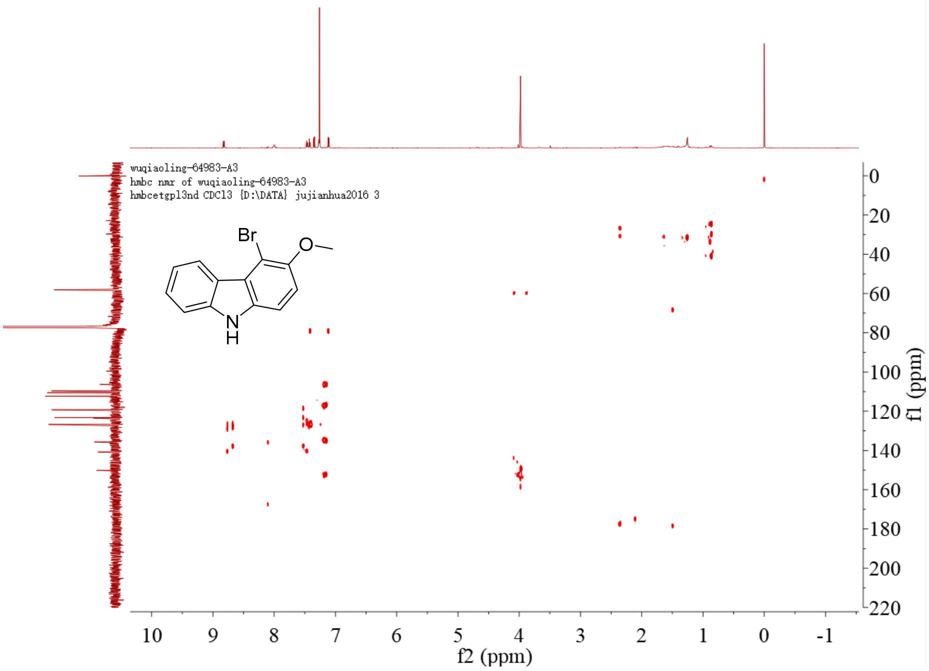


**Figure S38.** HMBC spectrum of compound **4** in chloroform-*d*.

## Mass Spectrum SmartFormula Report

**Analysis Info**

| | | | |
|---|---|---|---|
| Analysis Name | D:\Data\MS\data\202012\wuqiaoling_64983-A3_pos_11_01_9615.d | Acquisition Date | 12/31/2020 10:11:05 AM |
| Method | LC_Direct Infusion_pos_100-1000mz.m | Operator | SCSIO |
| Sample Name | wuqiaoling_64983-A3_pos | Instrument | maXis 255552.00029 |
| Comment | | | |

**Acquisition Parameter**

| | | | | | |
|---|---|---|---|---|---|
| Source Type | ESI | Ion Polarity | Positive | Set Nebulizer | 0.4 Bar |
| Focus | Active | Set Capillary | 4500 V | Set Dry Heater | 180 °C |
| Scan Begin | 100 m/z | Set End Plate Offset | -500 V | Set Dry Gas | 4.0 l/min |
| Scan End | 2000 m/z | Set Charging Voltage | 0 V | Set Divert Valve | Waste |
| | | Set Corona | 0 nA | Set APCI Heater | 0 °C |

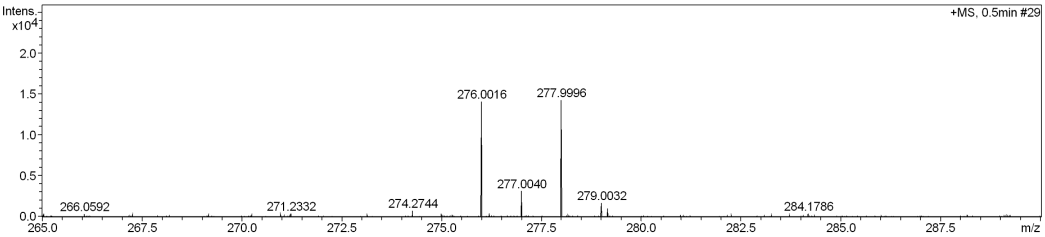


| Meas. m/z | # | Ion Formula | Score | m/z | err [ppm] | err [mDa] | mSigma | rdb | e⁻ Conf | N-Rule |
|---|---|---|---|---|---|---|---|---|---|---|
| 276.0016 | 1 | C13H11BrNO | 100.00 | 276.0019 | -0.9 | -0.2 | 36.6 | 8.5 | even | ok |

wuqiaoling_64983-A3_pos_11_01_9615.d

Bruker Compass DataAnalysis 4.1 printed: 7/28/2022 5:41:24 PM by: SCSIO Page 1 of 1

**Figure S39**. HRESIMS of compound **4**.

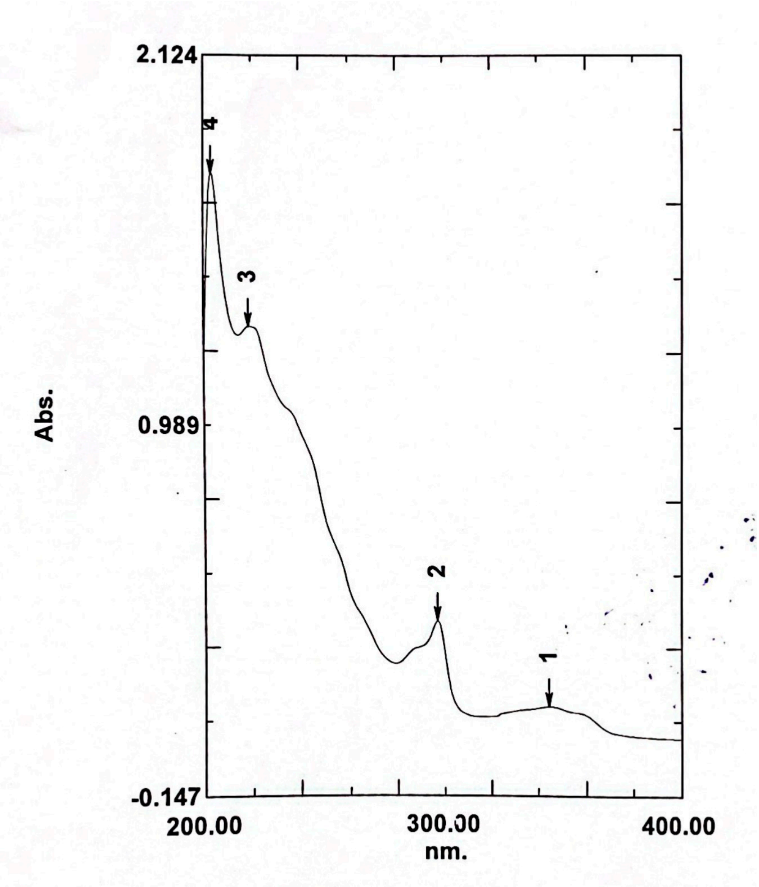


**Figure S40**. UV spectrum of compound **4**.

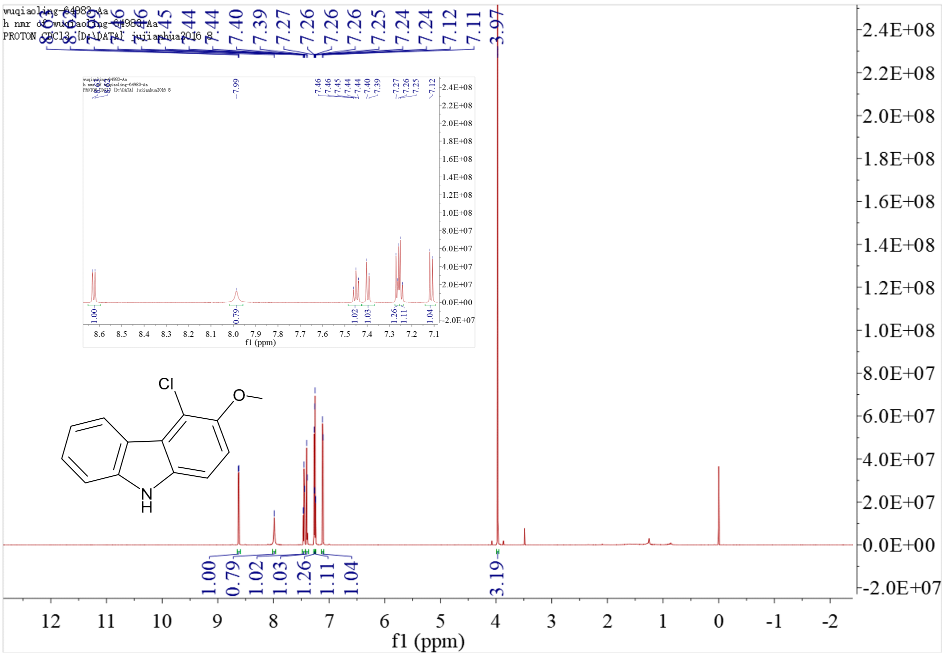


**Figure S41**.$^{1}$H-NMR spectrum of compound **5** in chloroform-*d*.

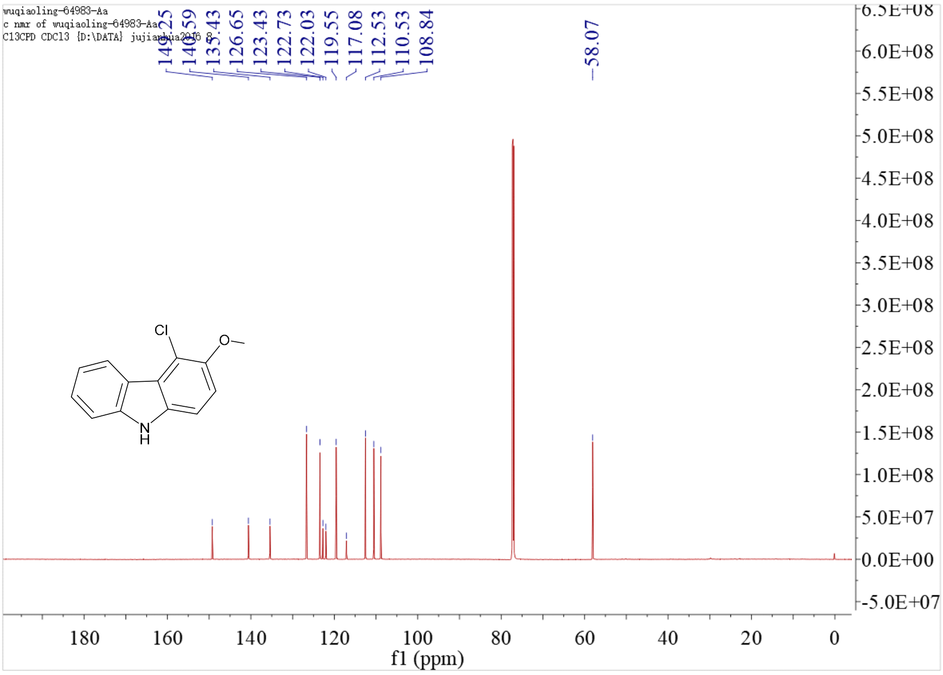


**Figure S42.**$^{13}$C-NMR spectrum of compound **5** in chloroform-*d*.

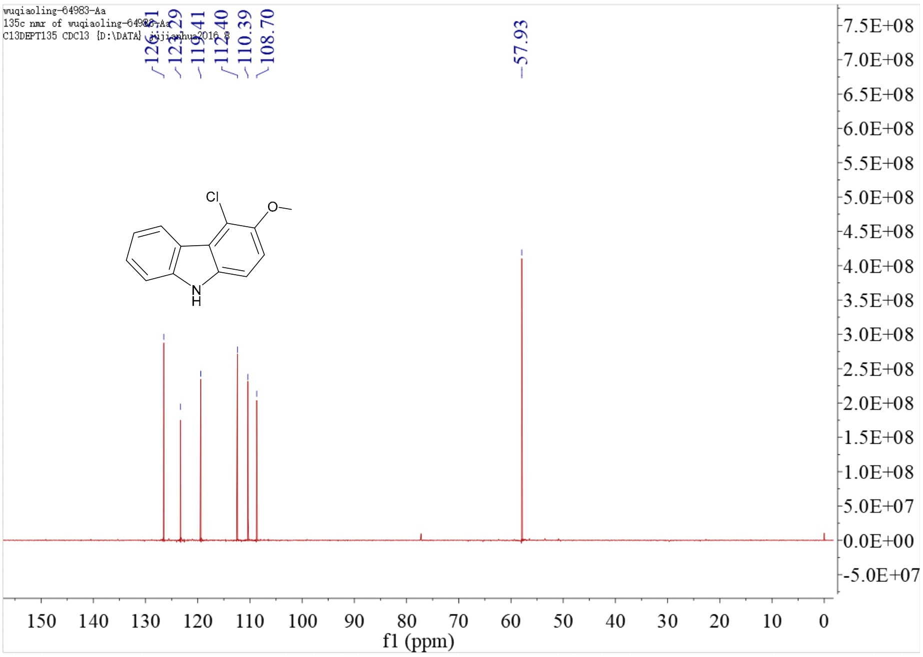


**Figure S43.** DEPT135 spectrum of compound **5** in chloroform-*d*.

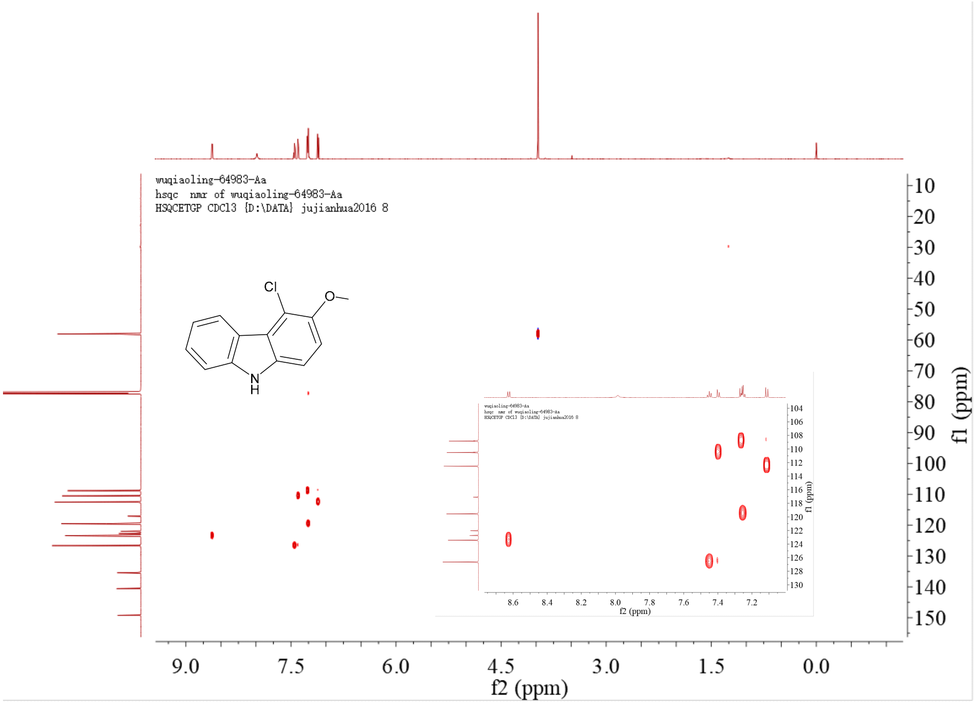


**Figure S44.** HSQC spectrum of compound **5** in chloroform-*d*.

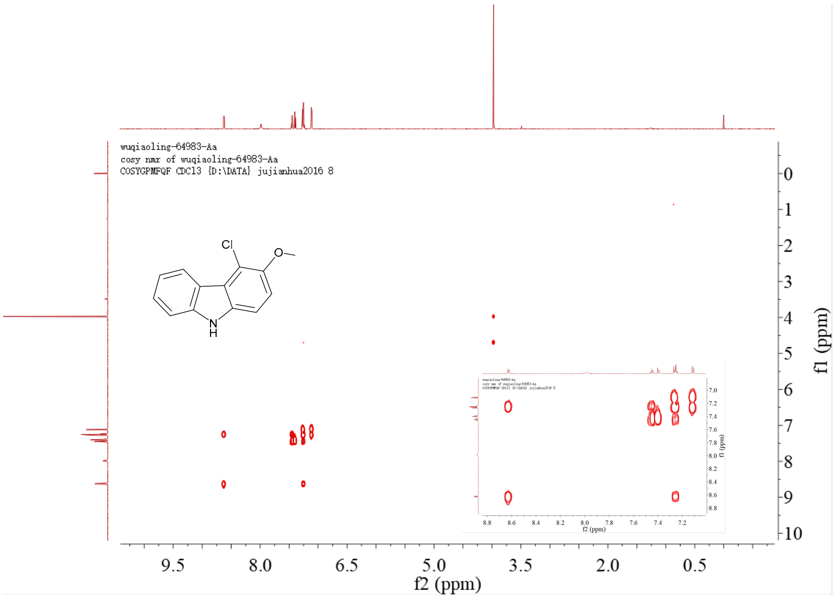


**Figure S45.**$^{1}$H-$^{1}$H COSY spectrum of compound **5** in chloroform-*d*.

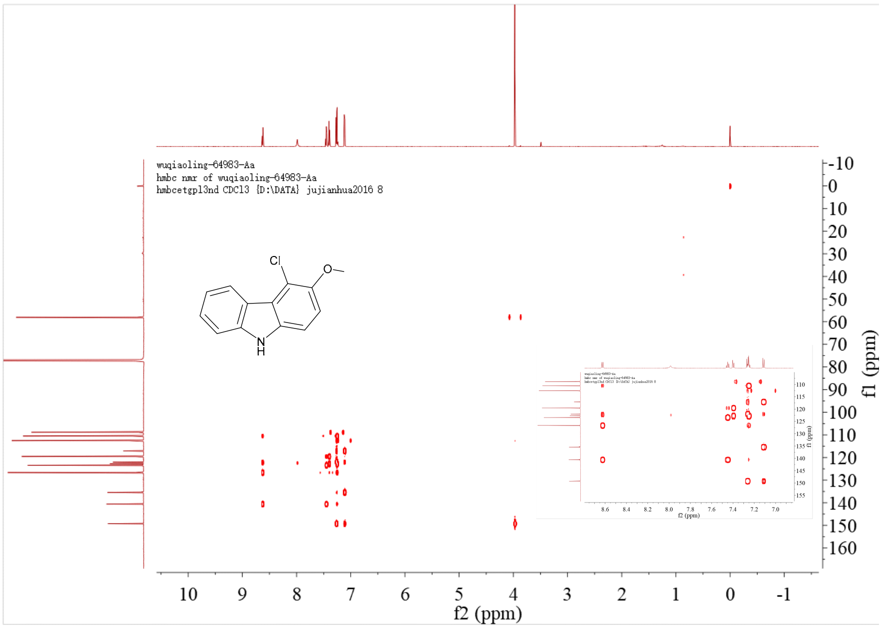


**Figure S46.** HMBC spectrum of compound **5** in chloroform-*d*.

## Mass Spectrum SmartFormula Report

**Analysis Info**

| | | | |
|---|---|---|---|
| Analysis Name | D:\Data\MS\data\202012\wuqiaoling_64983-A_pos_52_01_9437.d | Acquisition Date | 12/9/2020 3:21:49 PM |
| Method | LC_Direct Infusion_pos_100-1000mz.m | Operator | SCSIO |
| Sample Name | wuqiaoling_64983-A_pos | Instrument | maXis 255552.00029 |
| Comment | | | |

**Acquisition Parameter**

| | | | | | |
|---|---|---|---|---|---|
| Source Type | ESI | Ion Polarity | Positive | Set Nebulizer | 0.4 Bar |
| Focus | Active | Set Capillary | 4500 V | Set Dry Heater | 180 °C |
| Scan Begin | 100 m/z | Set End Plate Offset | -500 V | Set Dry Gas | 4.0 l/min |
| Scan End | 2000 m/z | Set Charging Voltage | 0 V | Set Divert Valve | Waste |
| | | Set Corona | 0 nA | Set APCI Heater | 0 °C |

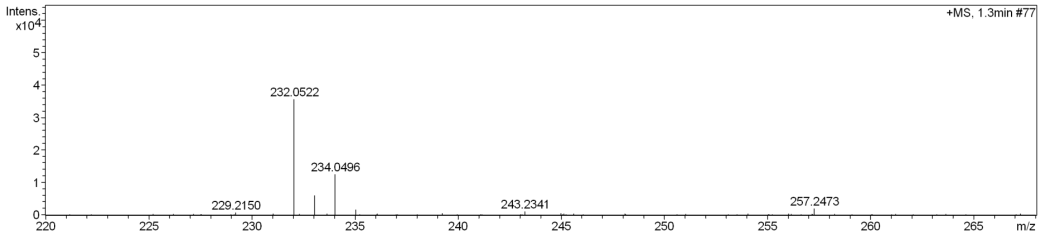


| Meas. m/z | # | Ion Formula | Score | m/z | err [ppm] | err [mDa] | mSigma | rdb | e⁻ Conf | N-Rule |
|---|---|---|---|---|---|---|---|---|---|---|
| 232.0522 | 1 | C13H11ClNO | 100.00 | 232.0524 | -0.8 | -0.2 | 15.0 | 8.5 | even | ok |
| 254.0355 | 1 | C13H10ClNNaO | 100.00 | 254.0343 | -4.7 | -1.2 | 230.3 | 8.5 | even | ok |

wuqiaoling_64983-A_pos_52_01_9437.d

Bruker Compass DataAnalysis 4.1 printed: 7/28/2022 5:41:58 PM by: SCSIO Page 1 of 1

**Figure S47.** HRESIMS of compound **5**.

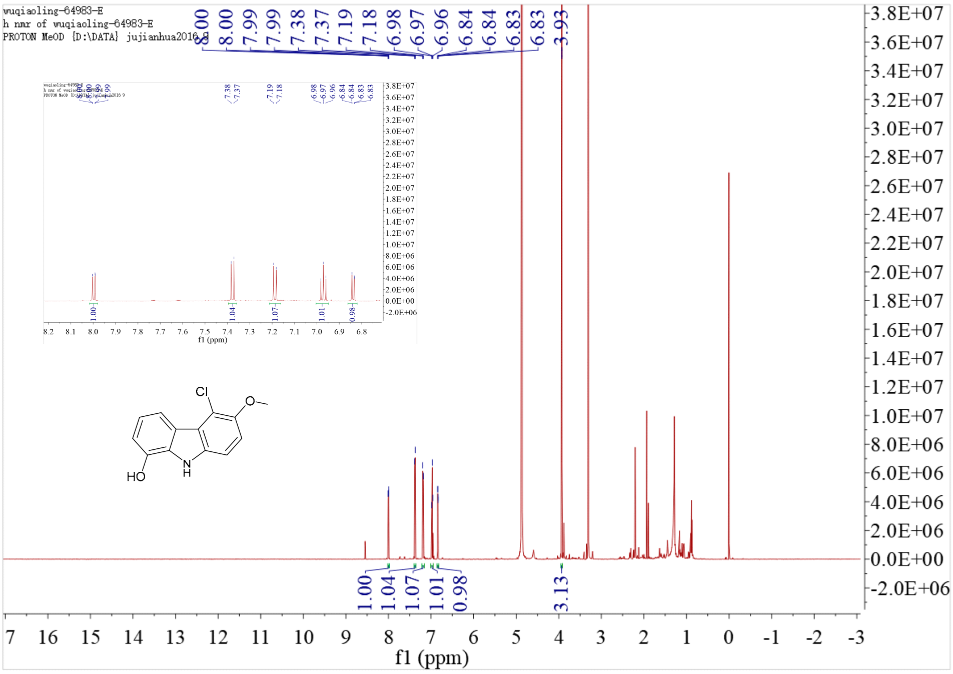


**Figure S48.** $^{1}$H-NMR spectrum of compound **6** in mathanol-*d*4.

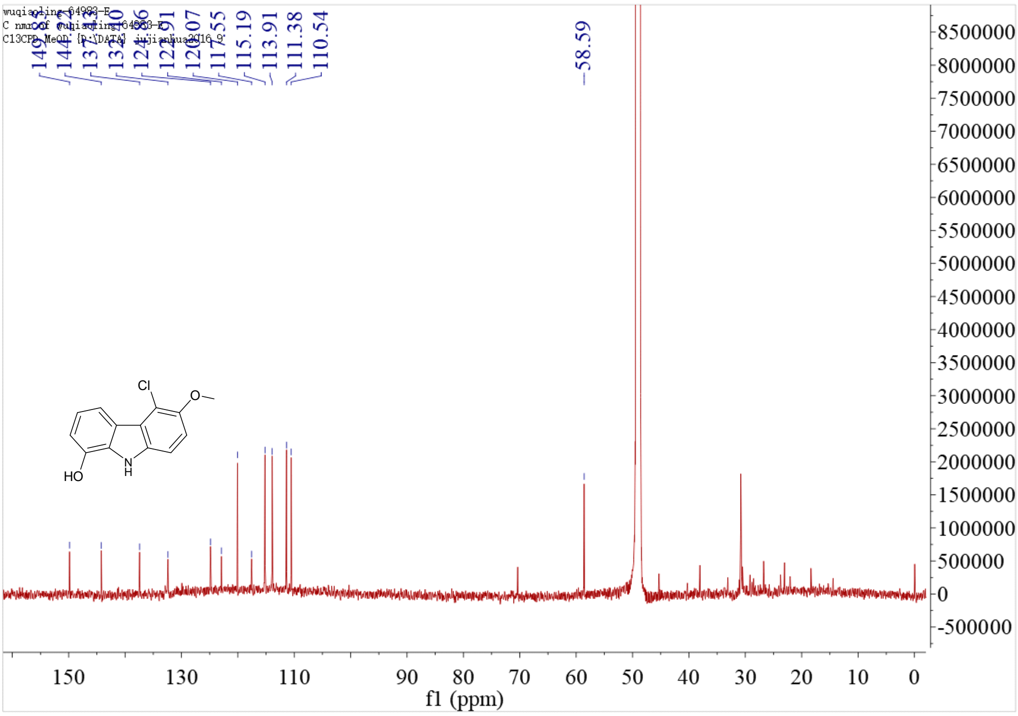


**Figure S49.** $^{13}$C-NMR spectrum of compound **6** in mathanol-*d*4.

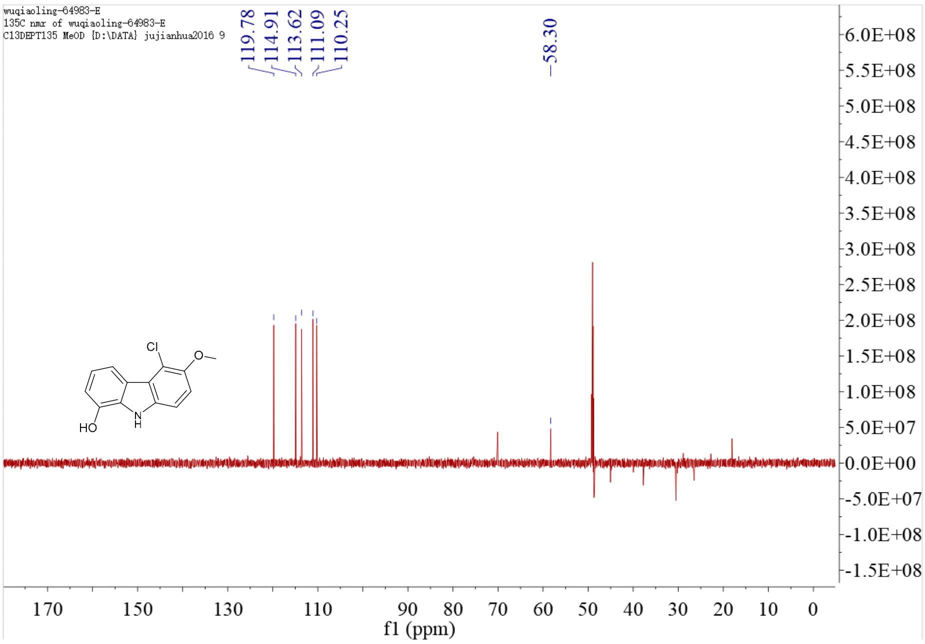


**Figure S50**. DEPT135 spectrum of compound **6** in mathanol-*d*4.

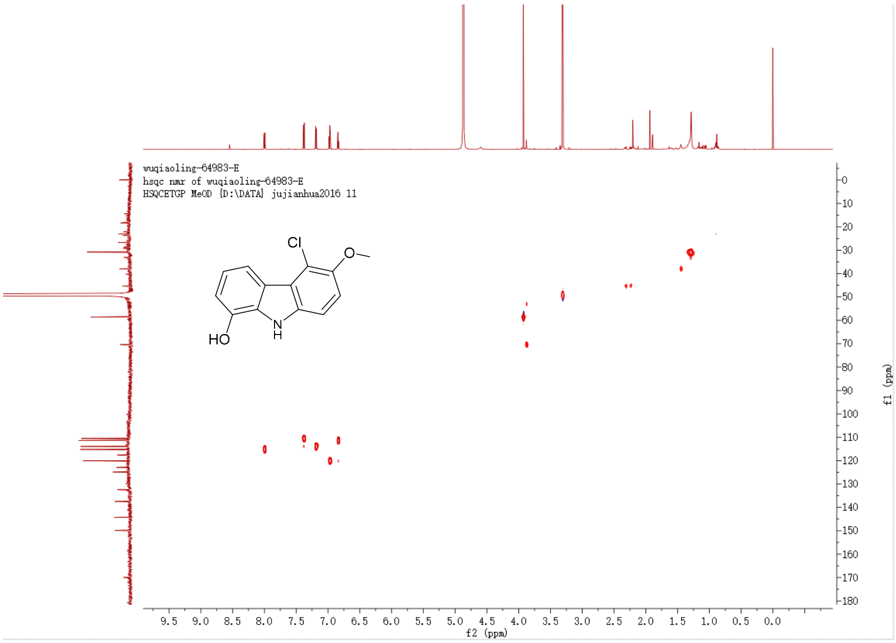


**Figure S51.** HSQC spectrum of compound **6** in mathanol-*d*4.

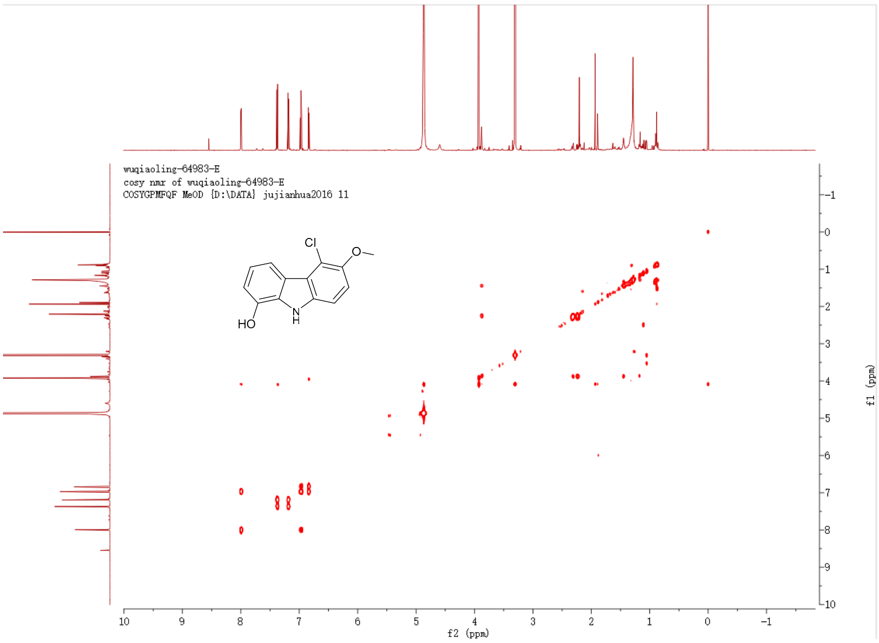


**Figure S52.** $^{1}$H-$^{1}$H COSY spectrum of compound **6** in mathanol-*d*4.

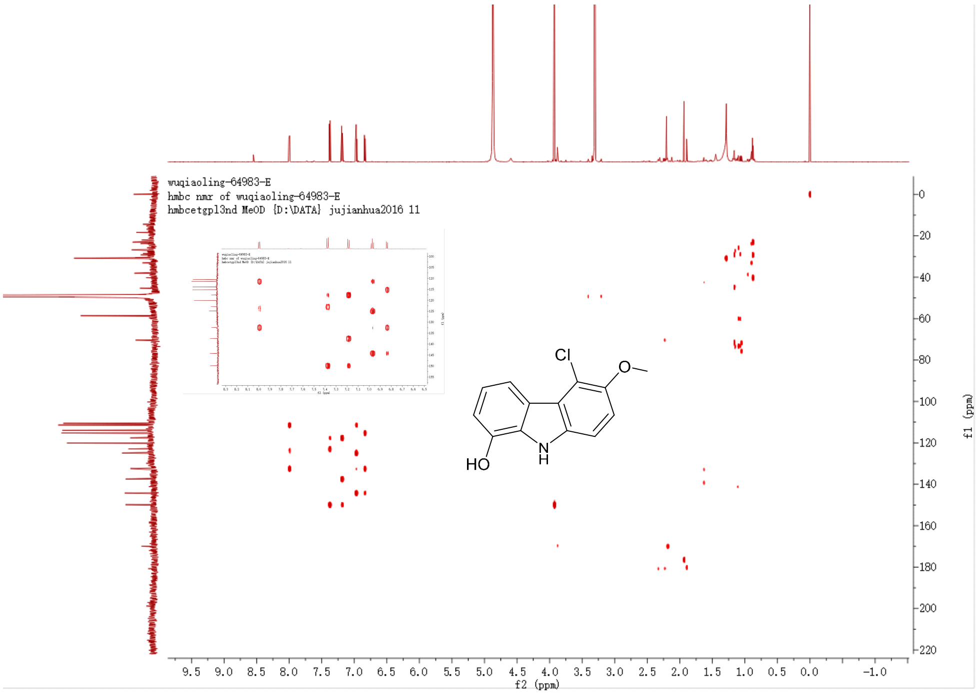


**Figure S53.** HMBC spectrum of compound **6** in mathanol-*d*4.

## Mass Spectrum SmartFormula Report

**Analysis Info**

| | | | |
|---|---|---|---|
| Analysis Name | D:\Data\MS\data\202105\wuqiaoling_64983-F_pos_64_01_10682.d | Acquisition Date | 5/11/2021 11:11:27 AM |
| Method | LC_Direct Infusion_pos_70-500mz.m | Operator | SCSIO |
| Sample Name | wuqiaoling_64983-F_pos | Instrument | maXis 255552.00029 |
| Comment | | | |

**Acquisition Parameter**

| | | | | | |
|---|---|---|---|---|---|
| Source Type | ESI | Ion Polarity | Positive | Set Nebulizer | 0.4 Bar |
| Focus | Active | Set Capillary | 4500 V | Set Dry Heater | 180 °C |
| Scan Begin | 70 m/z | Set End Plate Offset | -500 V | Set Dry Gas | 4.0 l/min |
| Scan End | 1500 m/z | Set Charging Voltage | 0 V | Set Divert Valve | Waste |
| | | Set Corona | 0 nA | Set APCI Heater | 0 °C |

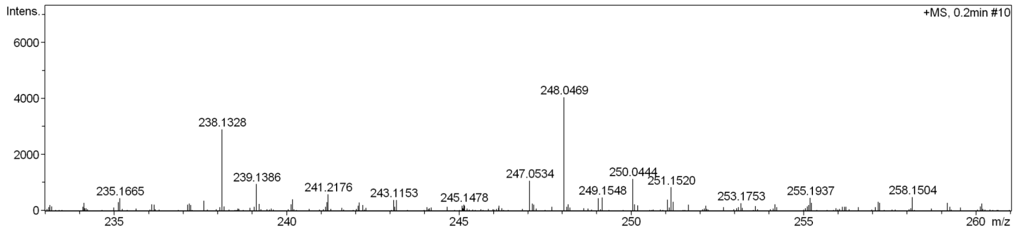


| Meas. m/z | # | Ion Formula | Score | m/z | err [ppm] | err [mDa] | mSigma | rdb | e⁻ Conf | N-Rule |
|---|---|---|---|---|---|---|---|---|---|---|
| 248.0469 | 1 | C13H11ClNO2 | 100.00 | 248.0473 | 1.4 | 0.3 | 36.1 | 8.5 | even | ok |

wuqiaoling_64983-F_pos_64_01_10682.d
Bruker Compass DataAnalysis 4.1 printed: 7/29/2022 9:10:43 AM by: SCSIO Page 1 of 1

**Figure S54.** HRESIMS of compound **6**.

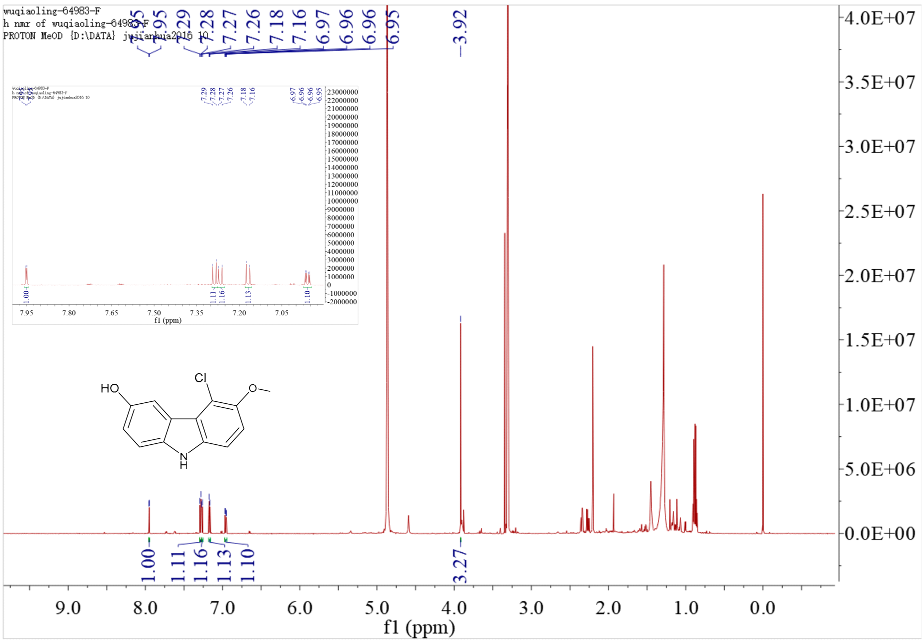

**Figure S55.**$^{1}$H-NMR spectrum of compound **7** in mathanol-*d*4.

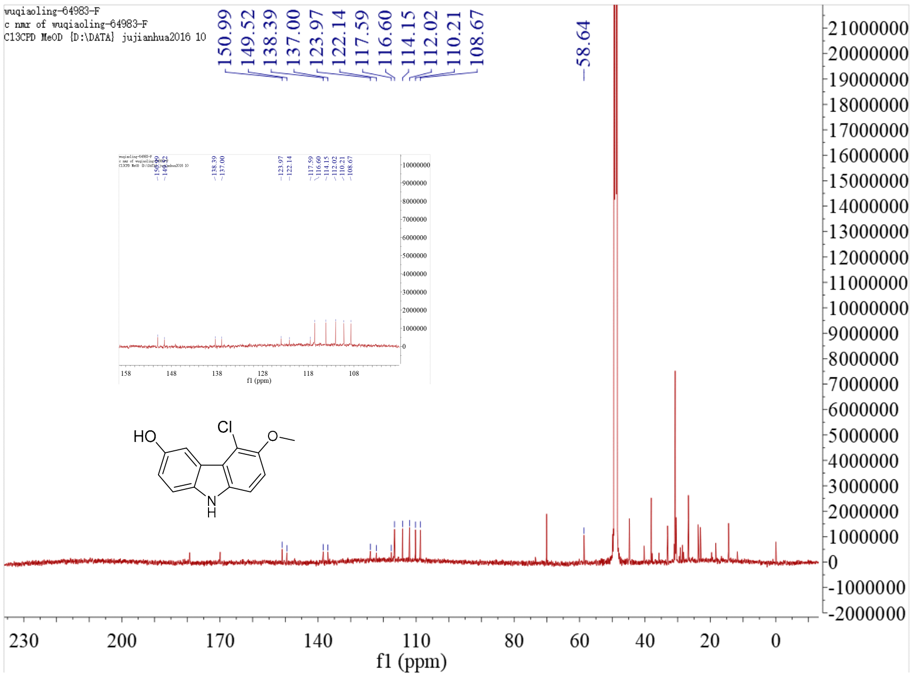

**Figure S56.**$^{13}$C-NMR spectrum of compound **7** in mathanol-*d*4.

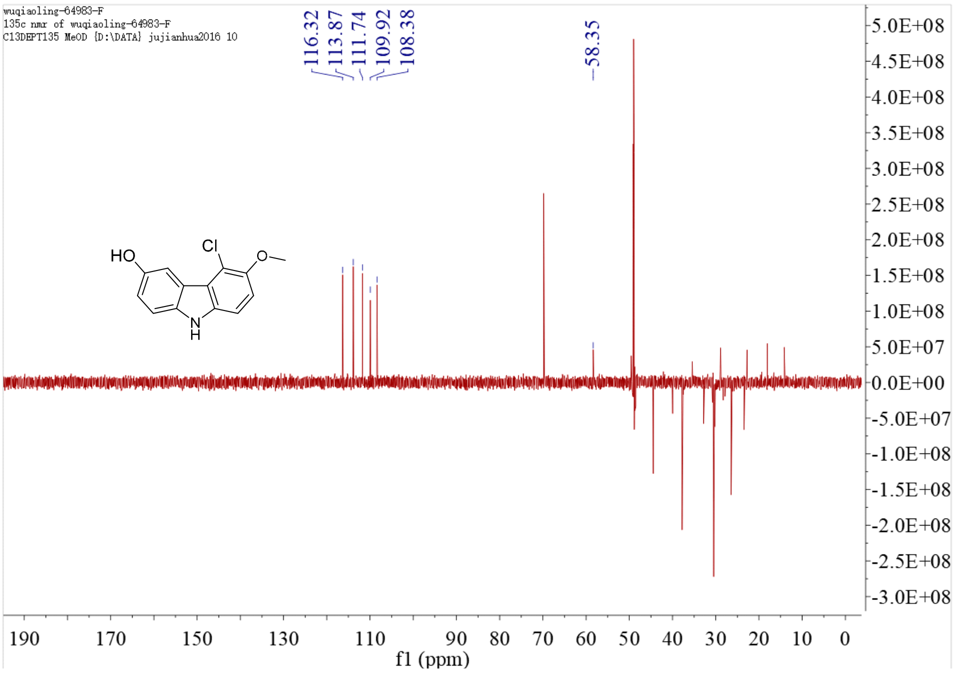


**Figure S57**. DEPT135 spectrum of compound **7** in mathanol-*d*4.

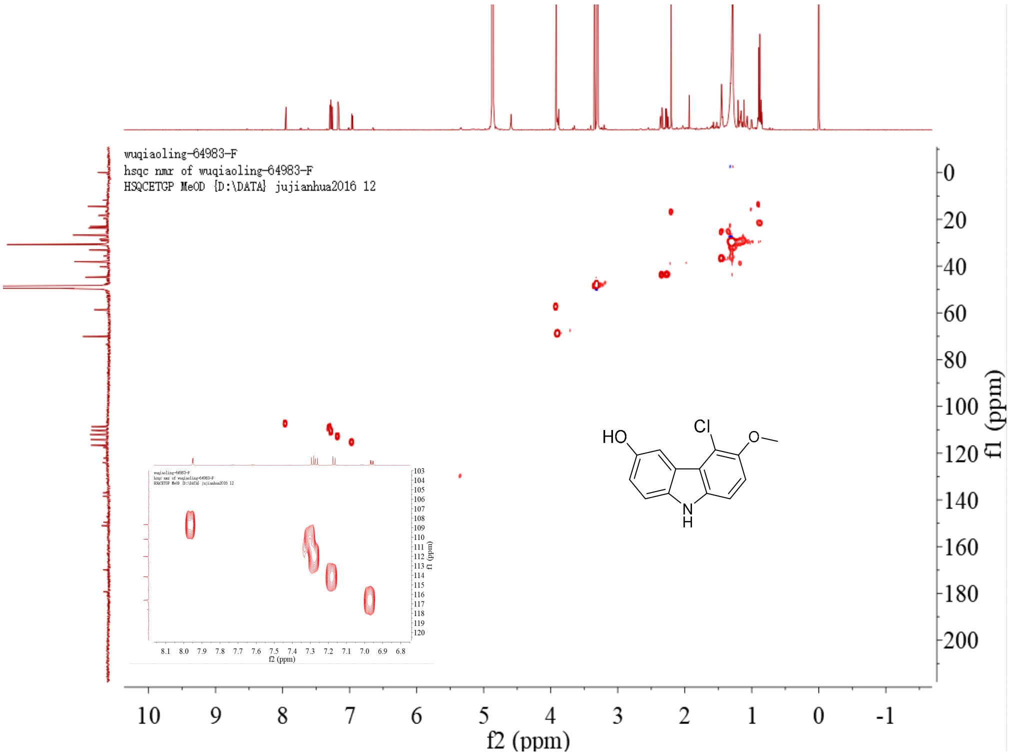


**Figure S58.** HSQC spectrum of compound **7** in mathanol-*d*4.

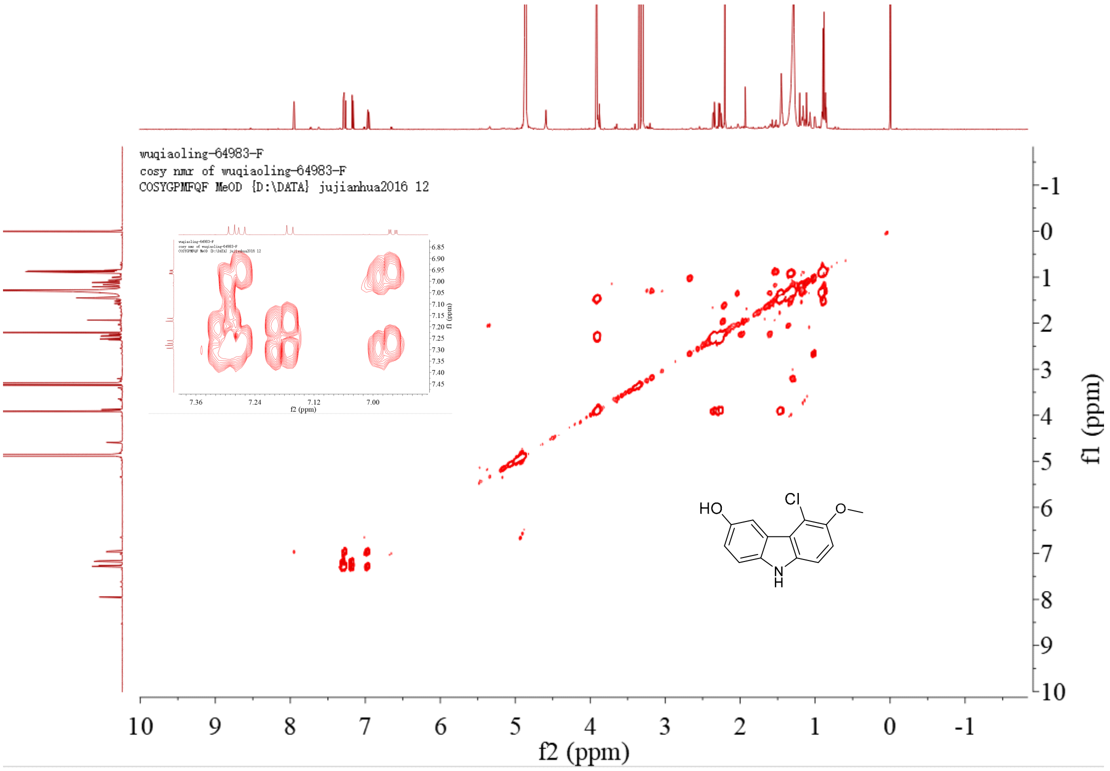


**Figure S59.**$^{1}$H-$^{1}$H COSY spectrum of compound **7** in mathanol-*d*4.

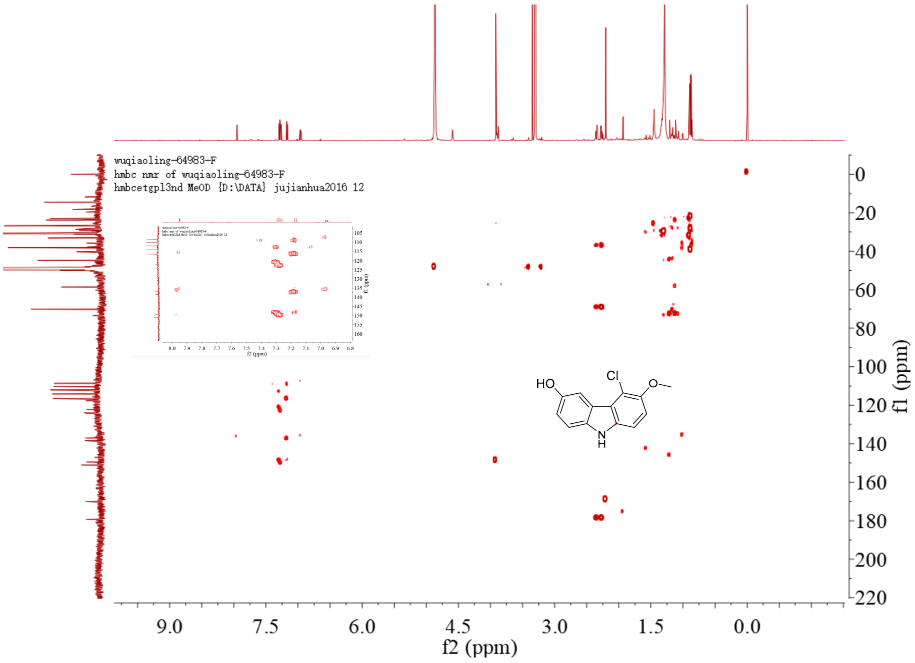


**Figure S60.** HMBC spectrum of compound **7** in mathanol-*d*4.

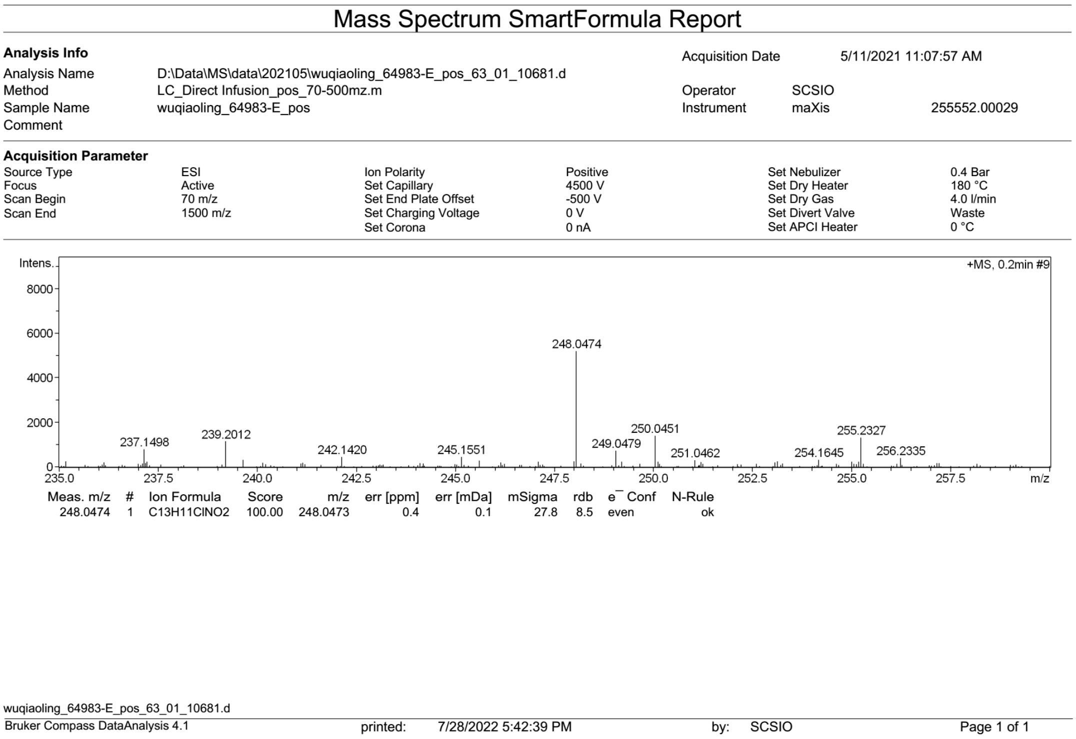
Mass Spectrum SmartFormula Report

| | | | |
|---|---|---|---|
| **Analysis Info** | | Acquisition Date | 5/11/2021 11:07:57 AM |
| Analysis Name | D:\Data\MS\data\202105\wuqiaoling_64983-E_pos_63_01_10681.d | | |
| Method | LC_Direct Infusion_pos_70-500mz.m | Operator | SCSIO |
| Sample Name | wuqiaoling_64983-E_pos | Instrument | maXis 255552.00029 |
| Comment | | | |

**Acquisition Parameter**

| | | | | | |
|---|---|---|---|---|---|
| Source Type | ESI | Ion Polarity | Positive | Set Nebulizer | 0.4 Bar |
| Focus | Active | Set Capillary | 4500 V | Set Dry Heater | 180 °C |
| Scan Begin | 70 m/z | Set End Plate Offset | -500 V | Set Dry Gas | 4.0 l/min |
| Scan End | 1500 m/z | Set Charging Voltage | 0 V | Set Divert Valve | Waste |
| | | Set Corona | 0 nA | Set APCI Heater | 0 °C |



| Meas. m/z | # | Ion Formula | Score | m/z | err [ppm] | err [mDa] | mSigma | rdb | e⁻ Conf | N-Rule |
|---|---|---|---|---|---|---|---|---|---|---|
| 248.0474 | 1 | C13H11ClNO2 | 100.00 | 248.0473 | 0.4 | 0.1 | 27.8 | 8.5 | even | ok |

wuqiaoling_64983-E_pos_63_01_10681.d
Bruker Compass DataAnalysis 4.1 printed: 7/28/2022 5:42:39 PM by: SCSIO Page 1 of 1

**Figure S61**. HRESIMS of compound **7**.

## 2. Medium Formula and Optimization Results of *S. diacarni* SCSIO 64983

In this experiment, 14 kinds of culture media were used for fermentation optimization. The specific medium formula is as follows：

N4: Starch 15 g/L, fish peptone 8 g/L, bacteriological peptone 5 g/L, glycerol 8 g/L, KBr 0.2 g/L, sea salt 30 g/L, calcium carbonate 2 g/L; pH: 7.2-7.4.

M11: Glycerol 30 g/L, Soybean powder 10 g/L, $CaCO_3$ 2 g/L, sea salt 30 g/L; pH: 7.2-7.4.

SCAS: Starch 40 g/L, Acid Hydrolyzed Casein Peptone 5 g/L, $KH_2PO_4$ 0.5 g/L, $MgSO_4 \cdot 7H_2O$ 0.5 g/L, $FeSO_4 \cdot 7H_2O$ 0.5 g/L; pH: 7.2-7.4.

HMT: Glycerol 20 g/L, fish powder 20 g/L, Yeast extract 5 g/L, $CaCO_3$ 5 g/L; pH: 7.2-7.4.

ISP2: Glucose 4 g/L, Yeast extract 4 g/L, Malt extract 10 g/L, sea salt 30 g/L; pH: 7.2-7.4.

ISP3: Oatmeal 20 g/L, Trace elements 1mL/L, $FeSO_4 \cdot 7H_2O$ 0.1 g/L, $MnCL_2 \cdot 4H_2O$:0.1 g/L, $ZnSO_4.7H_2O$ 0.1 g/L; pH: 7.2-7.4.

ISP4: Starch 10 g/L, Bacteriological Peptone 1 g/L, Yeast extract 5 g/L, $KH_2PO_4$ 1 g/L, $MgSO_4 \cdot 7H_2O$ 1 g/L, NaCl 1 g/L, $(NH_4)_2SO_4$ 2 g/L, sea salt 30 g/L, $CaCO_3$ 2 g/L, Trace elements 1mL/L; pH: 7.2-7.4.

AM2ab: Starch 15 g/L, Soybean powder 5 g/L, sea salt 30 g/L, Glucose 20 g/L, $MgSO_4 \cdot 7H_2O$ 0.5 g/L, $CaCO_3$ 2 g/L, Yeast extract 2 g/L, $KH_2PO_4$ 0.5 g/L, Bacteriological Peptone 2 g/L, NaCl 4 g/L; pH: 7.2-7.4.

RA: Glucose 10 g/L, Starch 20 g/L, Malt extract 10 g/L, sea salt 30 g/L, corn powder 5 g/L, maltose 10 g/L, $CaCO_3$ 2 g/L, Trace elements 100 μL; pH: 7.2-7.4.

AM3: Starch 15 g/L, Soybean powder 10 g/L, glycerol 15 g/L, Bacteriological Peptone 15 g/L, sea salt 30 g/L, $CaCO_3$ 5 g/L; pH: 7.2-7.4.

LB: TRYPTONE 10g/L, Yeast extract 5g/L, NaCl 10g/L; pH: 7.2-7.4.
YMS: Yeast extract 4g/L, Malt extract 10g/L, Starch 4 g/L, Oatmeal 8 g/L, sea salt 30 g/L; pH: 7.2-7.4.
MS: Soybean powder 20 g/L, Mannitol 20 g/L; pH: 7.2-7.4.
TSB: TSB finished powder 30 g/L; pH: 7.2-7.4.

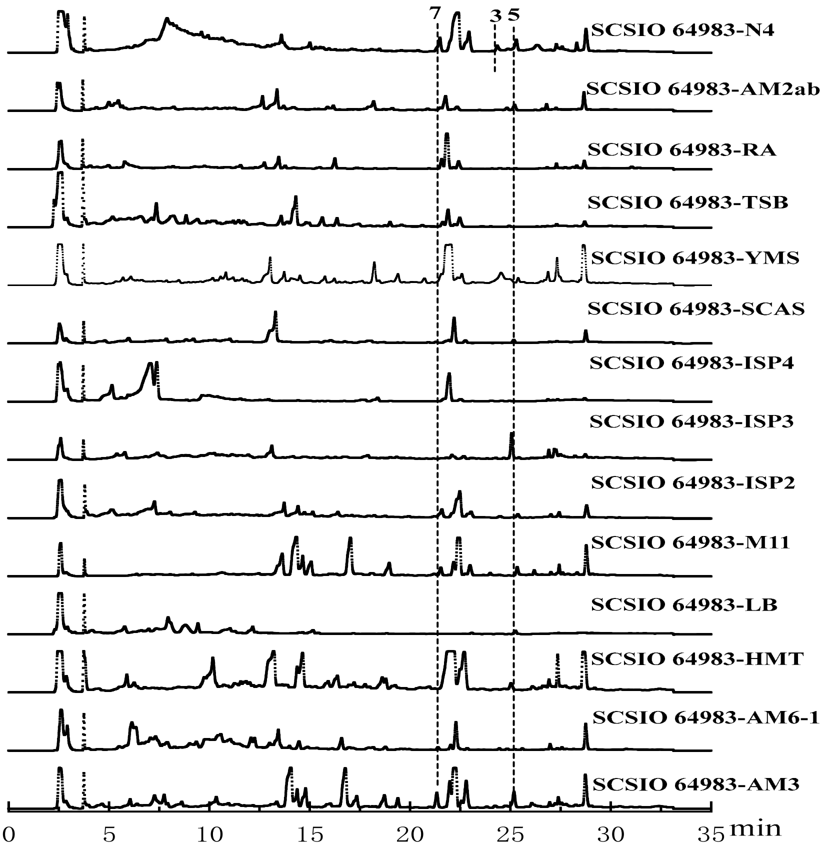


**Figure S62.** Optimization Results of *S. diacarni* SCSIO 64983

**3.** NMR data of **6-7** in methanol-$d_4$ and **1**–**2** in DMSO-$d_6$

**Table S1**. NMR data of **6-7** in methanol-$d_4$ and **1–2** in DMSO-$d_6$

| Position | 6[a] | | 7[a] | | 1[C] | | 2[C] | |
|---|---|---|---|---|---|---|---|---|
| | $\delta_C$, type | $\delta_H$ (J in Hz) | $\delta_C$, type | $\delta_H$ (J in Hz) | $\delta_C$, type | $\delta_H$ (J in Hz) | $\delta_C$, type | $\delta_H$ (J in Hz) |
| 1 | 110.5, CH | 7.38, d (8.7) | 110.1, CH | 7.29, d (8.7) | 111.0, CH | 7.49, d (8.1) | 110.1, CH | 7.42, d (8.7) |
| 2 | 113.9, CH | 7.19, d (8.7) | 114.1, CH | 7.17, d (8.7) | 125.6, CH | 7.40, m | 113.5, CH | 7.32, d (8.7) |
| 3 | 149.9, C | - | 149.5, C | - | 118.5, CH | 7.18, t (7.5) | 148.6, C | - |
| 4 | 117.6, C | - | 117.6, C | - | 122.0, CH | 8.28, d (7.9) | 113.6, C | - |
| 4a | 123.0, C | - | 122.0, C | - | 121.9, C | - | 120.3, C | - |
| 4b | 124.8, C | - | 123.8, C | - | 118.8, C | - | 118.5, C | - |
| 5 | 115.2, CH | 8.00, dd (0.7,8.0) | 108.6, CH | 7.95, d (2.4) | 112.6, C | - | 115.1, C | - |
| 6 | 120.1, CH | 6.97, t (7.8) | 150.9, C | - | 129.9, C | - | 130.1, C | - |
| 7 | 111.4, CH | 6.84, dd (0.7,7.6) | 116.6, CH | 6.95, dd (2.4,8.6) | 115.9, CH | 7.08, d (8.5) | 118.1, CH | 7.16, d (8.5) |
| 8 | 144.2, C | - | 112.0, CH | 7.27, d (8.6) | 109.2, CH | 7.33, d (8.5) | 109.7, CH | 7.34, d (8.5) |
| 8a | 132.5, C | - | 136.9, C | - | 135.9, C | - | 137.6, C | - |
| 9a | 137.5, C | - | 138.3, C | - | 140.2, C | - | 136.5, C | - |
| 1'' | - | - | - | - | 164.3, C | - | 165.8, C | - |
| 2'' | - | - | - | - | 29.0, $CH_2$ | 3.59, s | 30.4, $CH_2$ | 3.30, s |
| 3-OMe | 58.6, $CH_3$ | 3.93, s | 58.6, $CH_3$ | 3.92, s | - | - | 57.6, $CH_3$ | 3.87, s |
| 6-OMe | - | - | - | - | - | - | - | - |
| 9-NH | - | - | - | - | - | 11.37 | - | 11.59 |
| 10-NH | - | - | - | - | - | 10.52 | - | 10.37 |

[a] The data were recorded in methanol-$d_4$; [b] The data were recorded in chloroform-*d*; [C] The data were recorded in DMSO-$d_6$.
